# Virus-like structures for combination antigen protein mRNA vaccination

**DOI:** 10.1038/s41565-024-01679-1

**Published:** 2024-05-27

**Authors:** Jingjing Zhang, Yanmei Li, Fengyuan Zeng, Changyong Mu, Change Liu, Lichun Wang, Xiaowu Peng, Liping He, Yanrui Su, Hongbing Li, An Wang, Lin Feng, Dongxiu Gao, Zhixiao Zhang, Gang Xu, Yixuan Wang, Rong Yue, Junbo Si, Lichun Zheng, Xiong Zhang, Fuyun He, Hongkun Yi, Zhongshu Tang, Gaocan Li, Kaili Ma, Qihan Li

**Affiliations:** 1 Weirui Biotechnology (Kunming) Co., Ltd, Ciba Biotechnology Innovation Center, Kunming, China; 2 Shandong WeigaoLitong Biological Products Co., Ltd, Weihai, China

**Keywords:** Biomaterials, Nanobiotechnology

## Abstract

Improved vaccination requires better delivery of antigens and activation of the natural immune response. Here we report a lipid nanoparticle system with the capacity to carry antigens, including mRNA and proteins, which is formed into a virus-like structure by surface decoration with spike proteins, demonstrating application against SARS-CoV-2 variants. The strategy uses S1 protein from Omicron BA.1 on the surface to deliver mRNA of S1 protein from XBB.1. The virus-like particle enables specific augmentation of mRNAs expressed in human respiratory epithelial cells and macrophages via the interaction the surface S1 protein with ACE2 or DC-SIGN receptors. Activation of macrophages and dendritic cells is demonstrated by the same receptor binding. The combination of protein and mRNA increases the antibody response in BALB/c mice compared with mRNA and protein vaccines alone. Our exploration of the mechanism of this robust immunity suggests it might involve cross-presentation to diverse subsets of dendritic cells ranging from activated innate immune signals to adaptive immune signals.

## Abstract (web summary)

This paper presents a virus-like lipid nanoparticle decorated with spike proteins capable of carrying antigens, including mRNA and proteins, for vaccination against SARS-CoV-2 variants.

## Main

Since the global coronavirus disease 2019 (COVID-19) pandemic caused by severe acute respiratory syndrome coronavirus 2 (SARS-CoV-2) infection began at the end of 2019^1^, various vaccines against the disease have been developed and marketed^2^. However, the application of these vaccines did not completely constrain pandemic spread^3^, and the antibody levels found in epidemiological surveys have shown limited duration and low efficacy against viral variants^4^. These findings suggest that the interaction between SARS-CoV-2 and the host immune system is largely unknown, and the development of novel vaccines is needed^5^. Fortunately, breakthroughs in mRNA vaccines based on the successful development of lipid nanodelivery systems have provided new technical possibilities. Lipid nanoparticle (LNP) delivery of mRNA encoding an antigen into cells via fusion of the artificial lipid membrane and cellular plasma membrane results in effective expression of the viral spike protein (S protein) antigen in cells and induces subsequent presentation of the S antigen to activate innate/adaptive immunity via dendritic cells (DCs) and macrophages^6^.

According to viral immunological studies, the structural and/or non-structural antigens produced during viral replication in cells can be recognized by the cellular pattern recognition receptors of the innate immune system in the first event of antigenic signal stimulation of the host immune response. This implies that mRNA has the advantage of eliciting an immune response via such a natural route of immunity^7^; its strategy is, to some extent, similar to the infectious mechanism of the membranous virus SARS-CoV-2, although live viruses usually utilize the S protein in the viral membrane to bind specifically with the ACE2 receptor in the cellular membrane for viral genome internalization^8^. Therefore, when lipid delivery particles encapsulating mRNA molecules are loaded with S protein on their surface, these lipid particles might possess a virus-like structure (VLS) that enables targeting to cells via the receptor. In particular, the S protein of SARS-CoV-2 was found to interact with ACE2 or DC-SIGN receptors expressed on DC and macrophage surfaces^9,10^. These findings lead to the logical inference that if the proposed VLS system could be constructed, it would interact with not only the muscle cells or epithelium of injected local tissue but also the innate immune cells (for example, DCs and macrophages) carrying the ACE2 receptor or DC-SIGN^9,10^. Previously, an inhalable nanovaccine with a biomimetic coronavirus structure mimicking viral genetic material encapsulated by liposomes with receptor-binding domains (RBDs) of S protein loaded on the surface as ‘spikes’ to simulate the viral structure was reported, suggesting the possibility of VLS^7^. Therefore, the aim of this study was to design a VLS particle to encapsulate mRNA molecules and load proteins and to investigate its immunological characteristics, especially its capacity to elicit an immune response in a mouse model through surface S1 protein binding specifically to DC-SIGN on DCs and macrophages. The data obtained suggested that this strategy for delivering mRNA specifically into DCs through the VLS structure is very significant in comparison with regular mRNA vaccines.

## Results

### Characterization of VLS

**Fig. 1:**
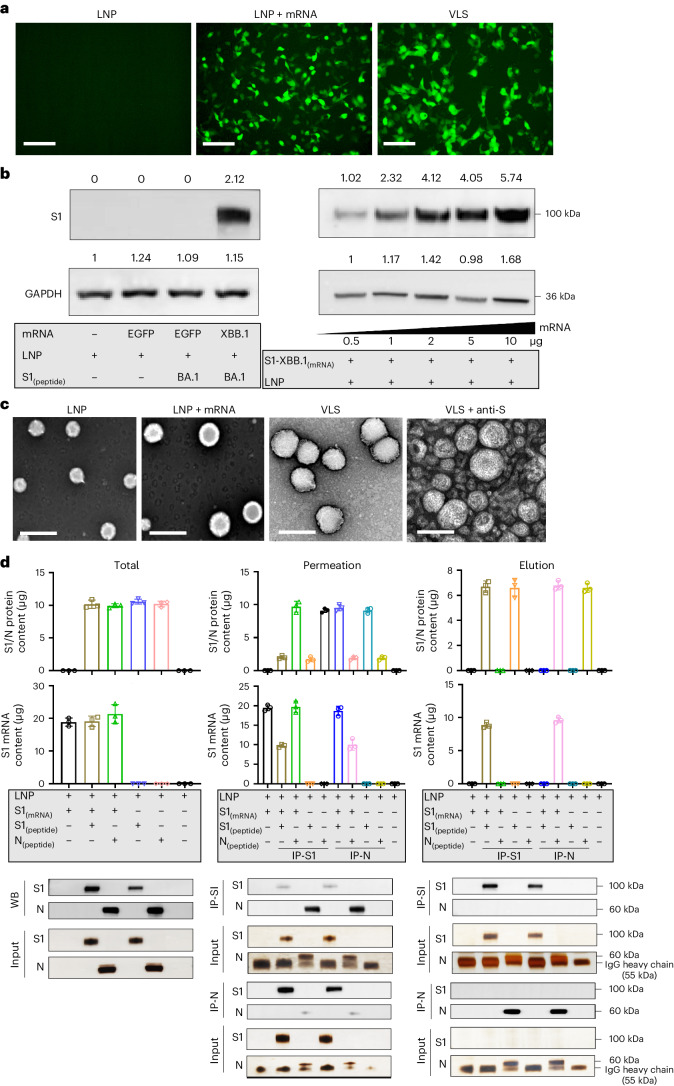
Characterization of VLSs and LNPs. **a**, Fluorescence micrographs revealed significant GFP expression for LNP, LNP encapsulating GFP mRNA and VLS. Scale bars, 250 μm. **b**, S1 proteins were assessed by Western blotting analysis of 293T cells transfected with LNPs or VLSs for 48 h. Left: S1 protein expression in 293T cells following transfection of either EGFP or S1 mRNA compared with LNPs; right: S1 protein expression in 293T cells following transfection with VLSs containing different doses of S1 mRNA. GAPDH was included as a protein loading control. The intensities of the signals (above the band) on Western blots were quantified by densitometric analysis with ImageJ (v.1.8.0.112). **c**, Electron micrographs of LNPs, LNPs encapsulating S1 mRNA, VLS and VLSs treated with a specific anti-S1 antibody. Scale bars, 200 nm. Experiments in **a**–**c** were replicated twice. **d**, Contents of protein (either S1 or N) and mRNA in total sample, penetration and elution. ‘IP-S1’ indicates that the samples were tested following incubation overnight with a specific anti-S1 antibody, and ‘IP-N’ indicates that the samples were incubated with an anti-N antibody. The experimental loading volumes were equal and identified by Western blotting and SDS‒PAGE silver staining (Input). The data are shown as the means ± s.d. from three independent experiments.

An important technical basis for the previous development of SARS-CoV-2 mRNA vaccines was the association of mRNA molecules with cationic lipids through static attraction and hydrogen bonding, which led to the encapsulation of mRNA molecules by LNPs^11^. In our VLS design, a lipid carrier encapsulating mRNA was required to load a surface protein, which might require a greater cationic lipid charge capacity for charge complexation in an ionic environment^12^. For lipid particles to acquire a greater capacity to carry mRNA and protein simultaneously, our design included two cationic lipid molecules, ((2-(2-hydroxyethoxyl)ethyl)azanddiyl)bis(hexane-6,1-diyl)bis(2-hexyldecanoate) and 1,2-dioleoyl-3-trimethylammonium propane; one central phospholipid, 1,2-dierucoyl-phosphatidylcholine; and one poly-polyethylene glycol (PEG) molecule, methoxypoly(ethylene glycol)-*N*-tetradecyltetradecanamide-1-2k. An LNP with this lipid composition and the mRNA encoding the SARS-CoV-2 XBB.1 strain S1 protein was produced through a microfluidic process (Supplementary Fig. 1a). The structural parameters of these characterized particles (Extended Data Table 1) and the identified mRNA molecules associated with the quantification of lipids (Supplementary Fig. 1a) suggest the typical features of mRNA vaccines. Moreover, this LNP was capable of delivering its encapsulated mRNA into 293 cells in a dose-dependent manner (Fig. 1a,b). Furthermore, the expressed and purified S1 recombinant protein (Supplementary Fig. 1a) was loaded on lipid particles encapsulating mRNA for the construction of VLSs with structural characteristics similar to those of mRNA vaccines (Extended Data Table 1). Our electron microscopy observations suggested that the loading process was irreversible (Supplementary Fig. 1b), and the morphological characterization of this VLS revealed particles with some bulges on the surface with differences compared to the LNP-encapsulated mRNA alone (Fig. 1c). Adding the S antibody enables the flocking of particles by antibody bridges (Fig. 1c). Structural analysis of the VLSs by immunoprecipitation with a specific S antibody suggested that the precipitated VLS particles contained mRNA molecules and S1 protein (Fig. 1d). These data support the use of VLS particles in our designed model.

### VLSs enable cell transfection and target ACE2/DC-SIGN molecules

**Fig. 2:**
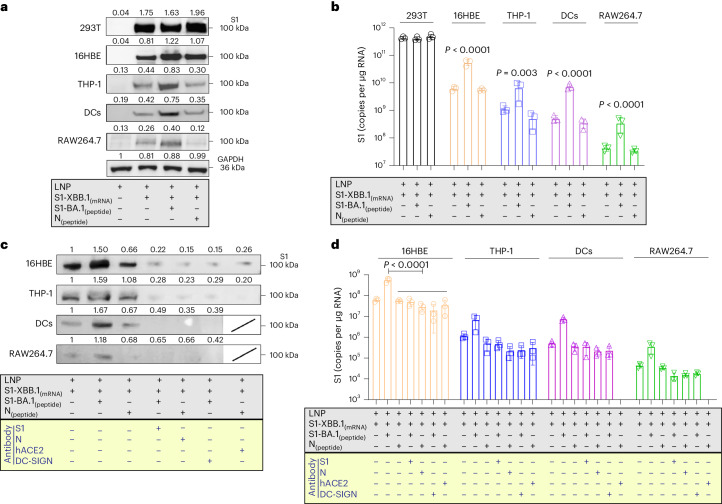
VLSs enhance the transfection of cells to achieve mRNA delivery through targeting ACE2/DC-SIGN molecules via the S1 protein. **a**, S1 proteins were expressed by Western blotting for different cells. **c**, S1 proteins were expressed by Western blotting which used antibodies to block receptors for different cells or S1 (N) protein for VLSs. LNPs and VLSs encapsulating S1 mRNA were incubated with 293T cells, 16HBE cells, THP-1 cells, DCs and RAW264.7 cells. Experiments in **a** and **c** were repeated twice. **b**, mRNA copies transfected into different cells were assessed by qRT-PCR. **d**, mRNA copies transfected into cells were assessed by qRT‒PCR which used antibodies to block receptors for cells or S1 (N) protein for VLSs. **b**, **d,** *n* = 3 independent samples, mean ± s.d. The blue text in the yellow frames shows the different antibody-treated samples or cells. Significant differences were determined using one-way analysis of variance (ANOVA) with Tukey’s post hoc test.

Compared with the LNPs of the mRNA vaccine, the VLSs improved the delivery capability of the mRNA when the protein was loaded on the surface. Binding to the ACE2 or DC-SIGN molecules of the S1 protein might result in VLSs targeting cells with both receptors in local tissue^12,13^. Our work indicated that VLSs were capable of not only transfecting 293 cells with high efficacy (Fig. 2a) but also of enhancing mRNA expression in cultured 16HBE cells, which showed not only copies of target mRNA (Fig. 2b) but also the expressed protein (Fig. 2a) and presented restitution with the addition of antibodies against S or ACE2 (Fig. 2c,d). The same was true for the THP-1 cells (Fig. 2a,b). Based on our identified data on approximately 16HBE cells expressing ACE2 and THP-1 cells expressing ACE2/DC-SIGN (Supplementary Fig. 2), the enhanced expression of mRNA in both cell lines is probably due to the mediation of ACE2 or DC-SIGN interactions with the S1 protein. Furthermore, treatment of THP-1 cells with a specific antibody against DC-SIGN also interfered with the expression of VLS mRNA (Fig. 2c,d). This suggested that VLSs enable the delivery of mRNA into human macrophages via S1 through interactions with ACE2 and DC-SIGN receptors, which is consistent with reported data^10^. Furthermore, mouse DCs (JASWIIs) expressing DC-SIGN were found to interact with the S1 protein (Supplementary Fig. 3a). Transfection of these cells with VLS-encapsulated EGF or S1 mRNA led to obviously greater mRNA expression than transfection with LNP-encapsulated mRNA alone, as the specific blocking of antibodies against S or DC-SIGN eliminated this effect (Fig. 2c,d and Supplementary Fig. 3c). The same experiment using mouse RAW264.7 macrophages with DC-SIGN also provided similar results (Fig. 2c and Supplementary Fig. 3b). Based on previously confirmed data showing that human DCs and macrophages were infected by SARS-CoV-2 due to their expression of ACE2/DC-SIGN^14,15^, our results suggested that the interaction of the VLS with ACE2 or DC-SIGN receptors might be involved in the activation of innate immunity.

### The interaction of VLSs with DCs and macrophages

**Fig. 3:**
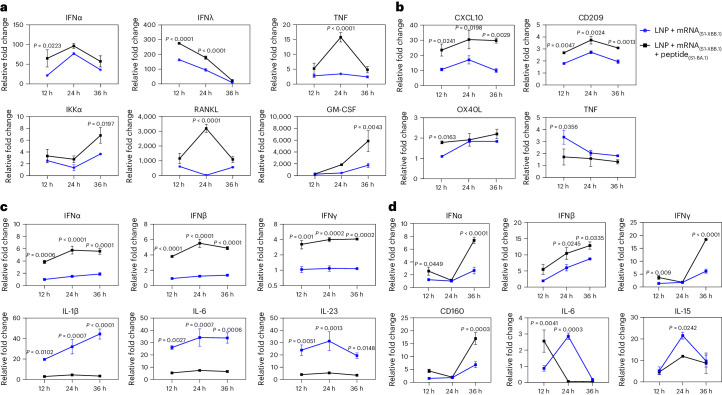
Transcription of immunological genes in cells transfected with LNPs and VLSs. **a**–**d**, Expression profiles of some major genes associated with the immune response in 16HBE (**a**), THP-1 (**b**), JASWII DC (**c**) and RAW264.7 (**d**) cells transfected with LNPs encapsulating only S1 mRNA or both S1 mRNA and S1 protein at 24 h after transfection. The blue represents LNPs encapsulating only S1 mRNA, and the black represents VLSs. The data are shown as the means ± s.d. from three independent experiments (*n* = 3). Significant differences were determined using one-way ANOVA with Tukey’s post hoc test.

Previous data suggested that the intense transcriptional response induced by LNPs to mRNA vaccines in cells was due to exogenous injury induced by LNPs and was thought to be an adverse reaction to mRNA vaccines^16–19^. We investigated whether a decrease in the activity of our VLS could be achieved through the binding of the S1 protein to the receptors. In our study, the expression of some molecules with immunological activation in innate/adaptive immunity tended to increase in 16HBE cells treated with VLSs (Fig. 3a), and increased expression of immune regulators and decreased expression of inflammatory mediators, such as tumour necrosis factor (TNF), were detected in the THP-1 cells (Fig. 3b). In addition, the expression of molecules, including TNF and GATA-3, tended to decrease in the JAWSII strain of mouse DCs, while the expression of members of the interferon (IFN) family tended to increase (Fig. 3c). A mild and balanced upregulation of the transcription profile of immune regulators and inflammatory factors was observed in RAW264.7 mouse macrophages (Fig. 3d). Interestingly, the upregulated expression of members of the interferon family in epithelial cells was similar to that in DCs and macrophages (Fig. 3a,c). These results suggest a certain immune activation role of VLSs in innate immunity that is different from that of mRNA/LNPs and support our hypothesis that the effect of the S1 protein on the VLS surface could lead to not only mRNA delivery but also activation of the DC-SIGN signalling pathway in DCs.

### Immunization of mice with VLSs elicits a discrepancy in immune signal transduction

**Fig. 4:**
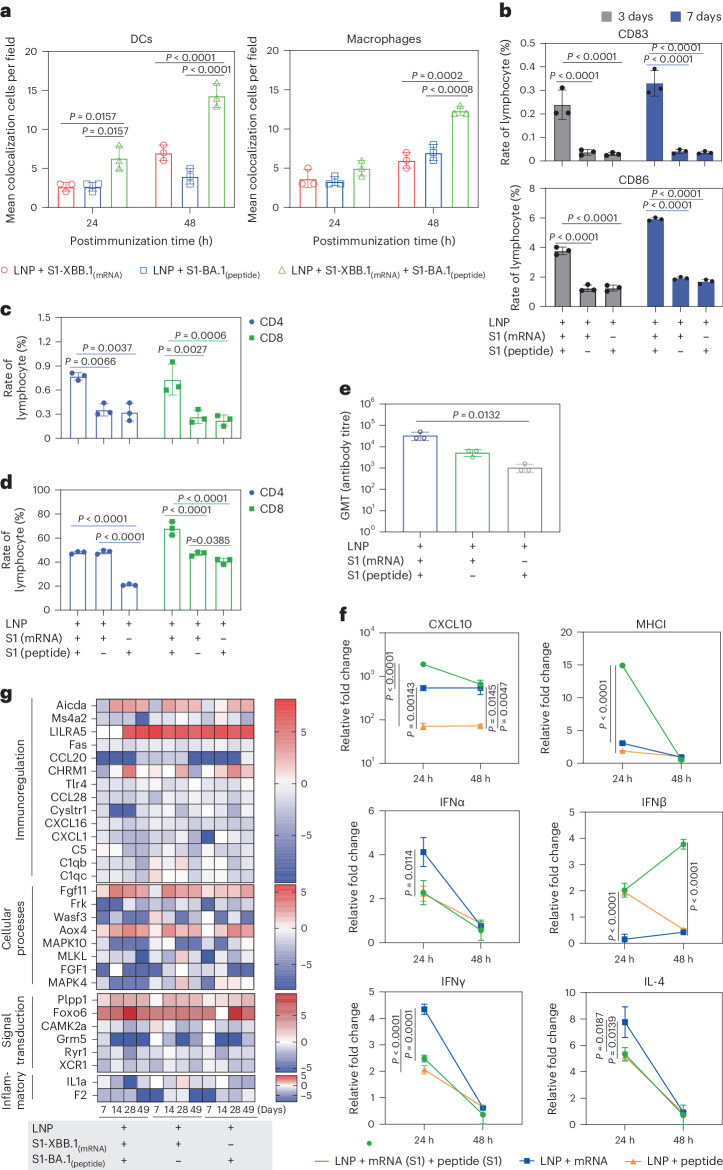
Innate immune responses were induced by VLSs in mice. **a**, Representative colocalization rates of S1 expression and DCs/macrophages in muscles of mice injected i.m. with VLSs, LNPs encapsulating S1 peptide or LNPs encapsulating S1 mRNA at 24 and 48 h. **b**, Ratio of CD86^+^ to CD83^+^ DCs in the spleens of mice after immunization with VLSs, LNPs encapsulating S1 peptide or LNPs encapsulating S1 mRNA for 3 days and 7 days. **c**,**d**, Ratio of specific memory T cells (**c**) and activated T cells (**d**) with marker molecules of CD8 in the spleen of the boosted and DC-treated transfusion VLSs, LNPs encapsulating S1 peptide or LNPs encapsulating S1 mRNA. **e**, Antibody titre in mice on day 21 after the transfusion of DCs from different treatment groups. **f**, Expression profiles at 24 and 48 h of some major genes associated with inflammation and the innate immune response in the muscle tissue of mice injected i.m. with VLSs, LNPs encapsulating S1 peptide or LNPs encapsulating S1 mRNA. **g**, Expression profiles of major genes associated with the activation or differentiation of immunoregulation, cellular processes, signal transduction and inflammation in lymph nodes at 7, 14 and 28 days after primary immunization and 21 days after booster immunization. The colour bars represent the log_2_ (fold change) compared with the value in the LNP group. The redder the colour, the more obviously the gene transcription level is upregulated. The relative expression levels of inflammatory cytokines in tissue were normalized to their levels in the blank control group (LNP) by using the comparative Ct (ΔΔCt) method. The data are shown as the means ± s.d. from three independent experiments (*n* = 3). Significant differences were determined using one-way ANOVA with Tukey’s post hoc test.

Our investigation of the interaction between VLSs and the immune system suggested that the mRNA encapsulated in the VLS, whether encoding GFP or the S1 protein, was expressed dynamically in local tissue (Supplementary Fig. 4), similar to what has been reported for mRNA vaccines^20^. Interestingly, the rate of S1 antigen colocalization with DCs or macrophages in local tissue in mice in the VLS group was greater than that in mice in the mRNA- or protein-alone groups (Fig. 4a and Supplementary Fig. 5a,b). The ratio of CD86^+^ DCs to CD83+ DCs, which suggested the maturity of DCs stimulated by toll-like receptor (TLR) signals, was greater in the spleens of mice in the VLS group on days 3–7 after primary immunization than in those in the mRNA or protein groups (Fig. 4b). The ratio of specific CD4^+^ to CD8^+^ memory T cells in the spleen was also greater in the boosted VLS group and in the group that received DCs pretreated with VLS than in the other two groups (Fig. 4c,d), which suggested that the interaction of VLSs with mouse DCs via S1 protein binding specifically to the DC-SIGN receptor followed by the effective expression of mRNA to augment the activation efficiency of DCs followed by subsequent activation of T cells was significant. Further antibody measurements revealed a greater antibody titre in mice that received DCs pretreated with VLSs on day 21 after injection than in mice that received DCs pretreated with mRNA or protein vaccines (Fig. 4e). Subsequent analysis of the transcription levels of various important immune molecules in the local tissue of mice injected with the experimental VLS vaccine between 24 and 48 h after immunization suggested increased expression of some functional molecules related directly to DCs and macrophage activation, such as CXCL10 and MHCI, compared with the expression found in mice immunized with mRNA or protein alone (Fig. 4f). The thousand-fold increase in CXCL10 suggested that the DCs activated by VLSs could be classified as cDCs^21^. Further Kyoto Encyclopedia of Genes and Genomes orthology transcriptomic analysis of lymphocytes collected from the spleens of immunized mice at various time points after immunization revealed a characteristic dynamic profile in the VLS group in comparison to that in the mRNA and protein groups (Fig. 4g). These variations in all transcriptional profiles suggested a discrepancy in host immune responses in individuals immunized with VLSs and mRNA or protein.

### VLSs elicit a more robust immune response in mice

**Fig. 5:**
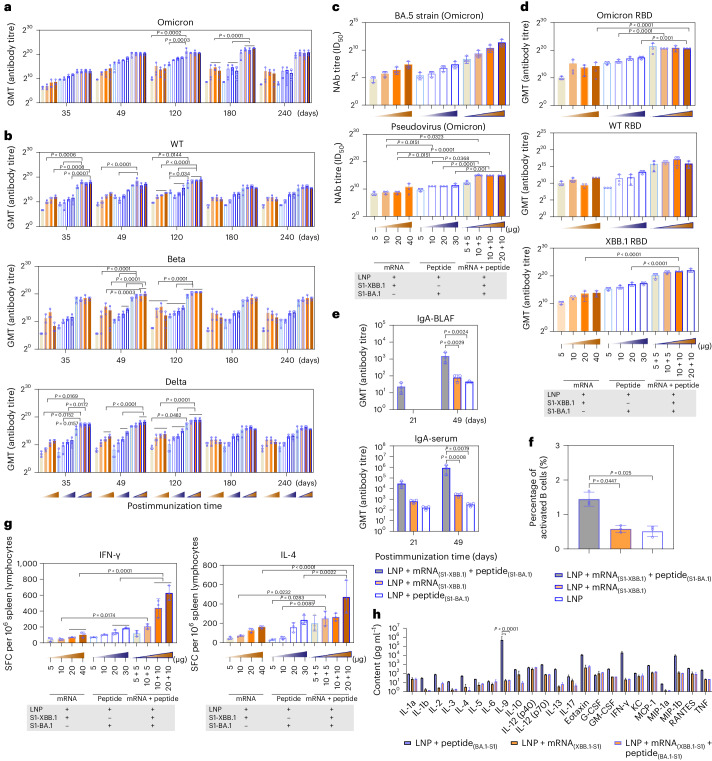
Adaptive immune responses were induced by VLSs in mice. **a**,**b**, SARS-CoV-2 titres of specific antibodies for Omicron (**a**) and wild-type (WT), Beta and Delta (**b**) strains whose production was induced by VLSs, LNPs encapsulating S1 peptide or LNPs encapsulating S1 mRNA in mice. The samples were obtained on days 35, 49, 120, 180 and 240 after primary injection. **c**, Titres of neutralizing antibodies (NAb) against the Omicron real virus strain BA.5 and the pseudovirus whose production was induced by VLSs, LNPs encapsulating S1 peptide or LNPs encapsulating S1 mRNA in mice. **d**, Titres of IgG antibodies against the RBD of the Omicron strain, XBB.1 strain and WT strain whose production was induced by VLSs, LNPs encapsulating S1 peptide or LNPs encapsulating S1 mRNA in mice. **e**, Titres of IgA antibodies against the spike protein of the Omicron strain whose production was induced by VLSs, LNPs encapsulating S1 peptide or LNPs encapsulating S1 mRNA in mouse BALF and serum 21 and 49 days after immunization. **f**, The activated B cells observed in lymph nodes of mice treated by VLS and mRNA vaccine. **g**, ELISpot responses of IFNγ and IL-4 in mice injected i.m. with VLSs, LNPs encapsulating S1 peptide or LNPs encapsulating S1 mRNA on day 49 after primary immunization. The orange frame represents LNPs encapsulating 5, 10, 20 and 40 μg S1 mRNA, from light to dark; the blue border represents 5, 10, 20 and 30 μg S1 peptide; and the orange frame in the blue border from light to dark represents LNPs encapsulating different combinations of RNA and peptide at different doses. SFC, spot-forming cells. **h**, Cytokines in the sera of mice immunized with VLSs, LNPs encapsulating S1 peptide or LNPs encapsulating S1 mRNA on day 28 after booster immunization. All assay values were calculated by subtracting the values detected with blank mouse serum. The data are shown as the means ± s.d. from three independent experiments (*n* = 3). Significant differences were determined using one-way ANOVA with Tukey’s post hoc test.

Based on the analysis of differences between our VLS vaccine and the mRNA vaccine (phase III clinical trial) currently used in the population (over 18 years old) (Supplementary Fig. 6), our immunological data suggested that the VLSs with mRNA and protein contents equal to those of the mRNA or protein vaccine elicited a robust immune response in immunized BALB/c mice, which had a much greater level of binding antibodies against the S antigen of the Omicron strain after immunization than that of mice immunized with mRNA or protein alone; the high level was maintained for more than eight months (Fig. 5a), and the mice showed obvious reactivity against S antigens of the Wuhan, Beta and Delta strains, with slightly lower levels (Fig. 5b). The synchronous detection of neutralizing antibodies against the variants with a pseudovirus and real virus also revealed similar results (Fig. 5c), in which neutralizing antibodies against the variants reached a level higher than 1:10^3^, which was confirmed to be effective for immune protection in previous studies of animals and humans^15,16^. The levels of antibodies against the RBD of the Wuhan, Omicron and XBB.1 strains were greater in the VLS group than in the other two groups (Fig. 5d). Compared with those in the other two groups, the levels of IgA secreted in the upper respiratory tract and in the serum were increased post-VLS immunization, especially after booster immunization, in the mice in the VLS group (Fig. 5e). Furthermore, the comparison of the ratio of activated B cells in the lymph nodes of mice in the VLS and mRNA groups indicated that the ratio of activated B cells in the VLS group was greater than that in the mRNA group (Fig. 5f). Importantly, an ELISpot assay specific for IFNγ and interleukin-4 (IL-4) showed a more intensive T-cell response in the VLS group than in the mRNA and protein groups on day 90 after immunization (Fig. 5g). Due to the obvious intensification of the IL-4 response observed, multiple cytokines in the sera of these mice were assayed at the same time, and the results indicated lower levels of most cytokines in the VLS group than in the mRNA or protein group (Fig. 5h). All the data suggested that the immune response elicited by VLSs should differ from that elicited by mRNAs or proteins based on the known activating effect of VLSs on innate immunity.

### Effective immune protection elicited in ACE^+/+^ mice immunized with VLSs

**Fig. 6:**
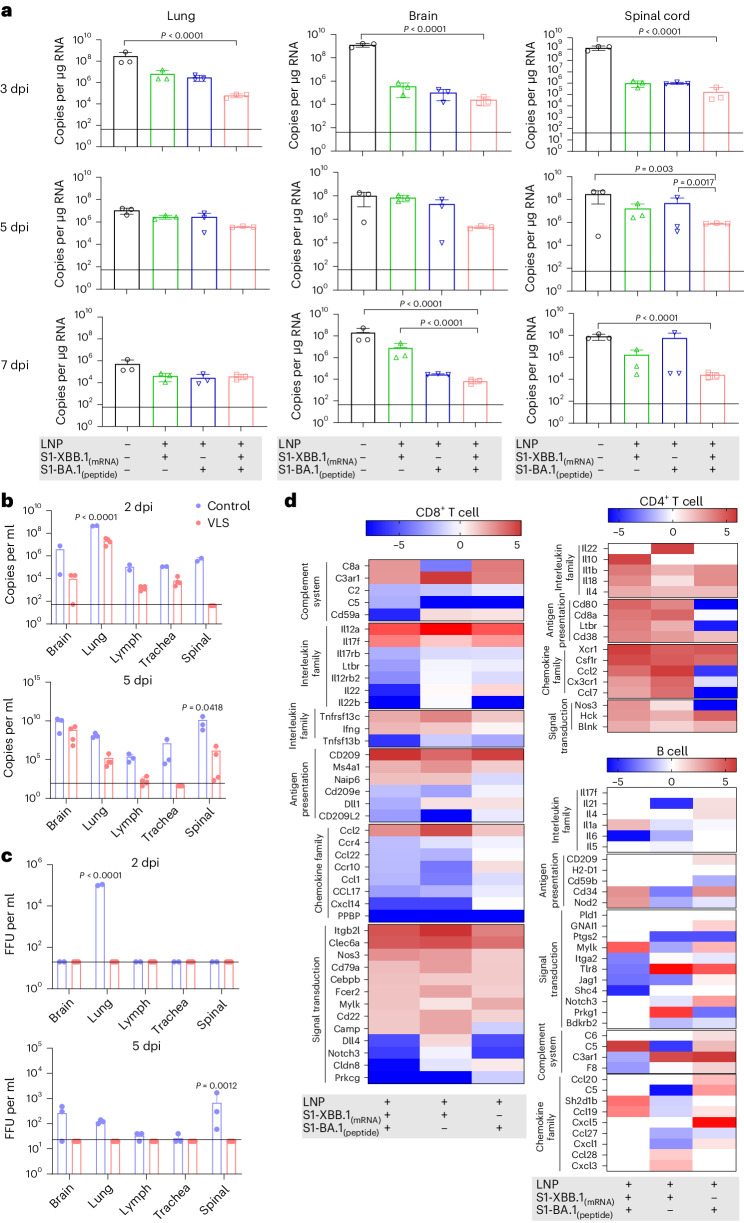
Effects of VLSs against Omicron BA.5 and XBB.1 strain challenge in C57-hACE^+/+^ mice. **a**, The viral loads in the lung, brain and spinal cord of hACE^**+/+**^ mice injected i.m. with VLS, LNPs encapsulating S1 peptide, or LNPs encapsulating S1 mRNA were evaluated following infection with the Omicron BA.5 strain, as determined by RT–qPCR. **b**,**c**, The viral loads in the brain, lung, lymph, trachea and spinal cord of hACE^+/+^ mice injected i.m. with VLSs and LNPs following infection with the Omicron XBB.1 strain were evaluated by RT–qPCR (**b**) and the viral titre was determined by virus plaque formation (**c**). Baseline was defined on the basis of the value detected in negative uninfected mice. FFU, focus forming unit; dpi, day(s) postinfection. **d**, Significantly differentially expressed genes associated with the activation or differentiation of CD8^+^ T, CD4^+^ T and B cells isolated from the lymph node tissue of mice injected i.m. with VLSs, LNPs encapsulating S1 peptide or LNPs encapsulating S1 mRNA 3 days after infection with the Omicron BA.5 strain. The colour bars represent the log_2_ (fold change) compared with the LNP group. A redder colour indicates more obvious upregulation of the gene transcription level. The data are shown as the means ± s.d. from three independent experiments (*n* = 3). Significant differences were determined using one-way ANOVA with Tukey’s post hoc test.

In our viral challenge test involving both ACE^+/+^ mice and hamsters (Supplementary Fig. 7), the results indicated that the VLSs completely protected the challenged mice from the Omicron BA.5 strain, XBB.1 and Wuhan strains because no symptoms were observed (Extended Data Table 2). Very little or no viral replication was detected in various organs of the mice during the infection period (Fig. 6a,b and Supplementary Figs. 8–10), and no histopathological changes were detected (Extended Data Table 3). Significantly, no live virus was detected in the plaque assay in any of the main organs of VLS-immunized mice challenged with XBB.1 in comparison to those of VLS-negative mice (Fig. 6c). In addition, the transcriptomic profiles of lymphocytes from BA.5-challenged mice were analysed on day 3. The results suggested that the differentially expressed genes related to the functional response of the immune system in the VLS group were obviously different from those in the other two groups (Fig. 6d). The categorization of B cells, CD4^+^ cells and CD8^+^ cells suggested that a greater percentage of DEGs were concentrated in the CD4^+^ cell and B-cell populations (Fig. 6d). These genes were mainly transcription-regulating molecules related to lymphocyte proliferation. Combined with the excellent immune protection efficacy in ACE^+/+^ mice in the VLS group, this unique transcriptional profile suggested an immunological outcome elicited by the integrative effect of diverse signals based on systematic activation of DCs under VLS stimulation through the robust binding of VLSs to DCs.

## Discussion

**Extended Data Fig. 1:**
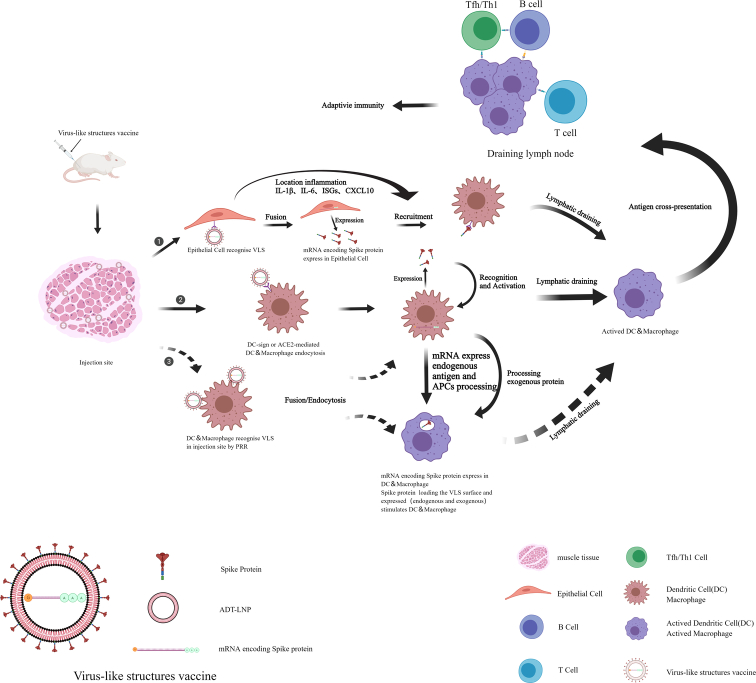
The speculated mechanism of VLSs vaccines interacting with host immune system and eliciting immune response in mice. ➀ Injected VLSs vaccines are endocytosed by the epithelial cells. After escaping from the endosome and entering the cytosol, mRNA is translated into protein by the ribosome. The translated antigenic protein can be secreted into the extracellular space. DCs and macrophages can be recruited by the local inflammation induced by VLSs. Both the secreted antigens and the antigens from the VLSs vaccine surface can be taken up by the recruited DCs and macrophages, followed by their activation and presentation to Tfh/Th1 and B cells. ➁ The injected VLSs vaccines are endocytosed by DCs and macrophages by the mediation of S1 protein binding specifically with DC-sign or ACE2 receptor. The delivered mRNA is translated into protein as an endogenous antigen that can activate DCs and macrophages. These activated immune cells enable antigen presentation to Tfh/Th1 and B cells for specific immunity activation and specific proliferation of B cells for antibody responses, while raising the specific CTL response. ➂ S1 protein antigen in the surface of VLSs vaccines, as an exogenous antigen, is recognized and endocytosed by DCs and macrophages, followed by their activation and antigen presentation to Tfh/Th1 and B cells. All antigenic signals from these pathways can activate and improve specific immune response.

During the past years of the COVID-19 pandemic, mRNA vaccines have provided us with novel vaccine research techniques and novel ideas for vaccine design and development^22–24^. This technological approach induces biological mimicry of infectious agents and offers new possibilities for the development of next-generation COVID-19 vaccines^25^. Based on this progress, our work established an improved lipid system that broadens the capacity of antigen carriage to include mRNA and protein. This system depends on the structure, which consists of mRNA and viral protein combined via specialized lipid particles assembled from two cationic lipids, one central phospholipid and one poly-PEG molecule. This lipid system was capable of efficiently encapsulating mRNA encoding the S1 antigen inside and carrying the S1 protein on its surface. Furthermore, our results revealed that the S1 protein on the surface enables binding with ACE2/DC-SIGN receptors to facilitate the entry of mRNA into cells and increase antigen expression, except for its expression via LNP fusion to the cell membrane. Therefore, we further showed that the S1 protein on the VLS surface plays an important role in guiding mRNA/LNPs to target DCs and macrophages, which results in significantly increased antigen uptake by the immune system. Further analysis of the transcription profiles of these cells suggested that the general trend in which VLSs were able to elicit the expression of multiple immune regulators and inflammatory mediators in a balanced way differed from that of mRNA/LNPs, which implied the significance of the VLS as a vaccine immunogen. This design was further verified by immunological analyses in vivo; we observed greater colocalization of S1 antigen and DCs or macrophages in local tissues and a greater percentage of activated DC populations in the lymph organs of individuals immunized with VLSs than in those immunized with mRNA/LNPs. Further investigation using BALB/c mice that received DCs pretreated with VLSs in vitro revealed a greater ratio of activated T cells in the VLS group than in the mRNA vaccine group. Importantly, mice immunized with the VLSs exhibited a more intense antibody response and stronger cytotoxic T-lymphocyte response than those immunized with the mRNA/LNPs. Interestingly, specific IgA secreted from the upper respiratory tract and in the serum was detected. Based on further protective animal tests, we found that such enhanced immunity provided more effective protection against Omicron BA.5, XBB.1 or the Wuhan strain. Further investigation revealed differences in the systemic upregulation of most genes in the CD4^+^ cell population and the upregulation of more than 50% of the genes in the B-cell and CD8^+^-cell populations in ACE^+/+^ mice in the VLS group upon challenge. These findings suggested a characteristic systematic immune response in mice immunized with the VLS, possibly because of the integrated effect of diverse signals from the innate immune system. These data support our speculation that VLSs enable interactions with various cells, which leads to not only endogenous antigen protein expression by mRNA and mRNA molecules recognized by intracellular TLRs^26^ but also the recognition of exogenous S1 protein by other TLRs in the plasma membrane of diverse subsets of DCs^27^, similar to binding to DC-SIGN or ACE2. Moreover, the antigenic chemotactic effect of epithelial cells transfected with VLSs via lipid fusion enabled the activation of DCs for antigen uptake and generated a certain antigenic signal. These events elicited during VLS immunization might lead to antigenic cross-presentation of diverse subsets of DCs and macrophages to T and B cells and could result in a robust immune response to VLS immunization (Extended Data Fig. 1). Therefore, we hypothesized that a strategy based on the specific induction of mRNA expression in DCs and macrophages could facilitate the development of virus-like vaccine candidates enabling the elicitation of an integrative immune response to provide more effective protection against viral variants than the current COVID-19 mRNA vaccine. However, this study has insufficient data to explain the physicochemical structure of the VLSs and the mechanisms underlying their interaction with cells (specifically DCs or macrophages), the signals elicited in these cells and the systematic immunological process in VLS-immunized individuals, which deserves further in-depth investigation.

## Methods

### Ethics statement

Four- to five-week-old K18-*hACE2* C57BL/6 mice (human *ACE2* gene knock-in C57BL/6 mice) by Cyagen Biosciences (animal license number SCXK (Su) 2022-0016). BALB/c mice and seven- to eight-week-old golden hamsters were purchased from Vital River (animal license numbers SCXK (Jing) 2021-0006 and SCXK (Jing) 2021-0011). All animals were bred in specific pathogen-fee barrier facilities (laboratory license number SYXK (Lu) 2020-0019). The laboratory animals were cared for and used following the ‘3 Rs’ principle and animal welfare guidelines. The animal experiment process and animal-related care and welfare were reviewed and approved by the Animal Experiment Ethics Committee of Shandong WeigaoLitong Biological Products (approval number LACUC-RD3-2022-006).

### Cell lines

HEK-293T and 16HBE cells were cultured in Dulbecco’s modified Eagle’s medium (DMEM, Thermo Fisher Scientific) supplemented with 10% fetal bovine serum (FBS; HyClone, GE Healthcare), 10% 100 U ml^−1^ penicillin and 100 mg ml^−1^ streptomycin. JASWII dendritic cells were cultured in MEM Alpha (Thermo Fisher Scientific) supplemented with 20% FBS and 5 ng ml^−1^ murine granulocyte–macrophage colony-stimulating factor (HY-P7361, MCE). RAW264.7 cells were cultured in minimum Eagle’s medium (MEM; Thermo Fisher Scientific) supplemented with 10% FBS. THP-1 cells were cultured in RPMI 1640 (Thermo Fisher Scientific) supplemented with 10% FBS and 0.05 mM β-mercaptoethanol (M917637, Macklin). All cells were purchased from ATCC and maintained at 37 °C with 5% CO_2_.

### Virus

The novel coronavirus H strain (Omicron BA.5) was isolated from COVID-19 patients in December 2022. The complete genome sequence was uploaded to NCBI GenBank (OQ179919.1). The novel coronavirus KMS-2 strain (Wuhan) was isolated from COVID-19 patients at the Hospital for Infectious Diseases in Yunnan Province in February 2020.

### Animal experimental design

C57BL/6-ACE2 (BALB/c or golden hamster) mice were randomly divided into five groups (Supplementary Fig. 7).

In group A (immune experimental group, *n* = 35), C57BL/6-hACE2 mice were divided into four groups: control, VLS, mRNA and peptide. The VLS contained 20 μg of mRNA and 10 μg of protein; the mice in the mRNA group were immunized with 20 μg of mRNA, and the mice in the peptide group were immunized with 10 μg of protein. All the mice underwent booster immunization on the 21st day after primary immunization. At 24, 48 and 72 h after primary immunization, tissues from the immune site were obtained for immunofluorescence detection for qRT–PCR measurement of cytokine expression. Moreover, B, CD4^+^ T and CD8^+^ T cells were sorted from lymph nodes at 3, 7 and 14 days after primary immunization and 28 days after booster immunization for transcriptome sequencing.

In group B (immune experimental group, *n* = 36), C57BL/6-hACE2 mice were divided into three groups: the control, VLS and mRNA groups. The mRNA used for the VLS and mRNA vaccines to explore the tissue site of mRNA molecules with the delivery system. The heart, liver, spleen, kidneys, lungs, brain and lymph nodes were collected at 1, 3, 5 and 7 days after i.m. injection for tissue immunofluorescence detection.

In group C (immune experimental group, n = 72), BALB/c mice were divided into 12 groups: N1–N12. N1–N4 were the mRNA vaccine groups, N5–N8 were the VLS vaccine groups and N9–N12 were the peptide vaccine groups. N1–N4 contained 5, 10, 20 and 40 μg mRNA, respectively. N5–N8 contained 5 μg mRNA and 5 μg peptide, 10 μg mRNA and 5 μg peptide, 10 μg mRNA and 10 μg peptide, and 20 μg mRNA and 10 μg peptide, respectively. N9–N12 contained 5 μg, 10 μg, 20 μg and 30 μg of peptide, respectively. All the mice underwent booster immunization on the 21st day after primary immunization. Blood samples were collected from the tail vein on days 35, 49, 120, 180 and 240 after immunization for antibody detection. At 28 days after booster immunization, blood was collected for neutralizing antibody and binding antibody testing, and the spleen was subjected to lymphocyte separation and ELISpot assays. At the same time, the immune titre of the SARS-CoV-2 vaccine was compared.

In group D (immunoprotective experimental group; C57BL/6-hACE2, *n* = 75; golden hamster, *n* = 40), at 28 days after booster immunization, the mice in the control, VLS, mRNA and peptide groups were challenged with SARS-CoV-2 Omicron BA.5 (10^4.5^ 50% cell culture infectious dose (CCID_50_)), SARS-CoV-2 wild-type (10^4^ CCID_50_) or SARS-CoV-2 Omicron XBB.1 (10^4.5^ CCID_50_) via the intranasal route. The heart, liver, spleen, kidneys, lungs, trachea, brain, spinal cord, lymph nodes and sex organs were collected at 3, 5 and 7 days after viral infection for viral load quantification. Meanwhile, B, CD4^+^ T and CD8^+^ T cells were sorted from C57BL/6-hACE2 mouse lymph nodes at 3 days after challenge for transcriptome sequencing.

In group E (transfusion experiment, *n* = 27), DCs were treated with LNPs-peptide, LNPs-mRNA and VLSs for 12 h. The in vitro-treated DCs (3 × 10^5^ cells per mouse) were transfused into mice through the tail vein. The spleen and inguinal lymph nodes were obtained at 3 and 5 days after adoptive transfer for flow cytometry and qRT‒PCR. Antibody titres were measured 21 days after transfusion. The above animal challenge experiments were commissioned by Wuhan Institute of Biological Products.

### Synthesis and formulation of the LNP delivery system

According to the molecular weights of ((2-(2-hydroxyethoxyl)ethyl)azanddiyl)bis(hexane-6,1-diyl)bis(2-hexyldecanoate), 1,2-dioleoyl-3-trimethylammonium propane, 1,2-dierucoyl-phosphatidylcholine and methoxypoly(ethylene glycol)-*N*-tetradecyltetradecanamide-1-2k, these were dissolved in absolute ethanol (Grade of guaranteed reagent) to molecular concentrations of 20%, 10%, 20% and 10%, respectively. All four components were mixed at a molar ratio of 30.68:6.84:15.2:1 in ethanol to prepare the LNP delivery system. The mRNA was diluted according to the requested ratio in buffer of 20 mM sodium acetate, 2.5 mM KCl and 0.1% trehalose. The LNP–mRNA was prepared with NanoAssemblr Ignite+ LNPs (Precision Nanosystems) by mixing blank LNPs with mRNA at a weight ratio of 1:3. The lipid particles were further characterized by Zetasizer Ultra spectrometry.

### Synthesis of SARS-CoV-2 S1 mRNA

The S1 sequence of SARS-CoV-2 was amplified and inserted into the PVAX vector by PCR technology. The 5′-untranslated region (UTR) of yellow fever virus was amplified and inserted before the Kozak sequence and the 3′-UTR of the human mitochondrial ribosome was amplified and inserted after the S1 sequence as the 5′-UTR and 3′-UTR of the S1 sequence. The constructed plasmid was linearized with BamHI (catalogue number R0136V, NEB) at 37 °C for 3 h for the in vitro transcription reaction. N1mψ-modified S1 mRNA was synthesized through an in vitro transcription reaction (HiScribe T7 ARCA mRNA Kit, catalogue number E2060S, NEB). The reaction mixture was treated with DNase I and purified using lithium chloride precipitation.

### Preparation of the VLS vaccine

The VLS vaccine was prepared by adding LNP–mRNA and peptide to the buffer (0.1% trehalose and 3.5% glucose) and mixing at room temperature for 30 min at 30 r.p.m. before rotating the mixture at 10 r.p.m. at 4 °C overnight. The VLS vaccine was further characterized by Zetasizer Ultra spectrometry.

### LNP mRNA- and VLS-transfected cells

Then, 0.5, 1, 2, 5 and 10 μg of mRNA was transfected into 293T cells with the LNP delivery system, and western blot detection was performed at 24 h after transfection. Meanwhile, the mRNA and VLSs were transfected into 16HBE, JASWII DCs, THP-1 cells and RAW264.7 cells for Western blot analysis and qRT‒PCR measurement at 24 h.

### Co-immunoprecipitation

Anti-S1 and anti-N antibodies were added to the LNP-encapsulated mRNA and VLS vaccine (the VLS vaccines were loaded with BA.1 S1 protein or N protein). Then, 50 μl of magnetic beads (BeaverBeads Protein A Matrix Antibody Purification Kit, catalogue number 20102, Suzhou) were added and incubated for 30 min at room temperature at 30 r.p.m. Next, the samples with magnetic beads were adsorbed at room temperature for 2–3 min at the magnetic pole, and the supernatant was used for mRNA and protein content measurement. Then, washing buffer was used to wash the beads, and elution buffer was added for elution and measurement of mRNA by qRT‒PCR and protein content by ELISA.

### qRT–PCR

The amount of mRNA in the VLS and mRNA vaccines was measured by qRT‒PCR. Based on the guidelines, the binding site designed for the primer was located in the SARS-CoV-2 S1 gene region, and part of the S1 gene was constructed in the pUC57 plasmid. In vitro-transcribed mRNA was used as a standard sample. The primers were F: TGGATCTGGAGGGAAAGCAGGGCAACT and R: CCGATTGGCAGATCCACCAGAGGTTC, and the TaqMan probe (Sangon Biotech) had the sequence 5′-6FAM-ATGGCTACTTCAAGATCTATAGCAAGC-TAMRA-3′.

The viral loads in the tissues were determined by qPCR with absolute quantification. Based on World Health Organization guidelines, the binding site designed for the primer was located in the SARS-CoV-2 N gene region, and the N gene was constructed on the pUC57 plasmid. The primers were F: GAATGGCTGGCAATGGCGGTGATGCT and R: TTGTTGGCCTTTACCAGACATTTTGCT, and the TaqMan probe (Sangon Biotech) had the sequence 5′-6FAM-TTGCTGCTGCTTGACAGATT-TAMRA-3′. Viral genomic RNA was extracted from tissues using RNAiso Plus (T9108, Takara), and the reactions were performed using a One Step PrimeScript RT‒PCR Kit (Perfect Real Time) (TaKaRa, code RR064A).

For the relative expression of cytokines in tissues and cells, total RNA was extracted with RNAiso Plus (T9108, Takara). Gene expression was expressed as the fold change (2^−∆∆Ct^) relative to the levels in samples from LNP-injected mice or virus-uninfected cells, which were used for calibration. The reactions were performed by using a One-Step SYBR PrimeScript PLUS RT–PCR Kit (TakaRa, code RR096A). The specific primers used are listed in Extended Data Tables 4 and 5.

### Immunofluorescence and confocal microscopy

Muscle tissues were collected and immediately frozen in liquid nitrogen. The tissue sections were embedded in OCT (Tissue-Tek OCT Compound 4583, Sakura) and sliced on a cryostat to a thickness of 5 µm (CM1850, Leica). Tissue sections were fixed and blocked with 5% BSA. For detection of the viral antigen, the sections were sequentially incubated with a primary mouse anti-SARS-CoV-2 S antibody (Sino Biological, catalogue number 40150-V08B1) and an Alexa Fluor 647-conjugated goat anti-mouse IgG secondary antibody (Invitrogen). DCs were detected using an anti-CD11c antibody (Abcam, catalogue number ab33483-N418), and macrophages were detected using an anti-F4/80 antibody (Zhengneng, catalogue number 263101-31B1), an Alexa Fluor 488-conjugated goat anti-rabbit IgG secondary antibody and a 647-conjugated goat anti-mouse IgG secondary antibody (Invitrogen). All cell nuclei were detected using 4,6-diamidino-2-phenylindole and analysed using a confocal microscope (TCS SP2, Leica).

### Virus titration

The virus titre was determined with a plaque assay in accordance with standard protocols, as described previously^28^. In brief, the virus was subjected to gradient dilution and added to six-well plates. Carboxymethylcellulose was used as the matrix in a liquid overlay, crystal violet was used as the stain to enhance plaque visualization, and the cells were cultured at 37 °C with 5% CO_2_ for 7 days.

### Neutralization assay

Briefly, serum was inactivated for 30 min at 56 °C, continuously diluted from 1:4 and mixed with virus at a titre of 100 times the CCID_50_/100 μl and incubated at 37 °C for 2 h. The mixture was added to a six-well plate and used carboxymethylcelluloseas the matrix, and crystal violet was used as the stain to enhance plaque visualization after culture at 37 °C with 5% CO_2_. Meanwhile, the serum neutralization titres of pseudoviruses were measured, and the 50% inhibitory dilution (EC_50_) was defined as the serum dilution at which the relative light units were reduced by 50% compared with those of the virus control wells (virus + cells) after subtraction of the background relative light units in the control groups with cells only. In brief, pseudovirus (Acro, catalogue number PSSO-HLC016) was incubated with serial dilutions of the test samples continuously diluted from 1:64 in duplicate for 1 h at 37 °C, together with the virus control and cell control wells. Then, 293T-ACE2 cells were added to each well. Following 48 h of incubation in a 5% CO_2_ environment at 37 °C, the luminescence was measured.

### IFNγ-specific and IL-4-specific ELISpot assay

For the ELISpot assay, mouse IFNγ and IL-4 ELISpot kits (Mabtech) were used according to the manufacturer’s protocol. Briefly, a plate was conditioned and seeded with splenic lymphocytes prior to the addition of 3 μg of stimulant (XBB Spike RBD Protein, catalogue number 40592-V08H144, Sino Biological; B.1.1.529 Spike RBD Protein, catalogue number SPD-C522e, Acro; BA.4/BA.5 Spike RBD Protein, catalogue number SPD-C522R, Acro; BA.2.12.1 Spike RBD Protein, catalogue number SPD-C522q, Acro; BQ.1.1 Spike RBD Protein, catalogue number SPD-C5240, Acro; BA.2.75.2 Spike RBD Protein, catalogue number SPD-C522z, Acro). Then, the cells were added and incubated at 37 °C for 30 h. Next, the cells and medium were removed, and the plate was developed. The coloured spots were counted using an ELISpot reader (Mabtech).

### Isolation of B, CD4^+^ T and CD8a^+^ T cells from mouse lymph nodes

Lymph nodes were isolated under sterile conditions and gently ground, and lymphocytes were separated into a suspension.

B cells were enriched with MajoSort Mouse CD19 Nanobeads (catalogue number SPD-C522R, Acro 480002, BioLegend), CD4^+^ T cells were enriched with the EasySep Mouse CD4^+^ T-cell isolation kit (catalogue number SPD-C522R, Acro 19852A, Stemcell), and CD8^+^ T cells were enriched with the EasySep Mouse CD8a^+^ selection kit (catalogue number SPD-C522R, Acro 18953, Stemcell).

### Transcriptome analysis of B, CD4^+^ T and CD8a^+^ T cells

The B, CD4^+^ T and CD8a^+^ T cells were sorted by lymph nodes. Total RNA was extracted using TRIzol (catalogue number DP421, Tiangen). RNA quantity and integrity were evaluated using a NanoDrop system and a Bioanalyzer, and the samples were prepared according to Illumina’s instructions and sequenced (Gene Denovo Biotechnology). Genes with 2-fold or greater changes in expression at *P* < 0.05 in the Kyoto Encyclopedia of Genes and Genomes analyses were selected and grouped into functional categories. All transcriptome sequencing results were uploaded to GSA; the assigned accession number of the submission was CRA010542.

### ELISA

The S1 antigen was quantified using a SARS-CoV-2 Spike Protein ELISA Kit (Acro, catalogue number RAS-A039), and the nucleoprotein was quantified using a SARS-CoV-2 (2019-nCoV) Nucleoprotein ELISA Kit (Jiya Biotechnology,). S1-RBD IgG assays were performed using SARS-CoV-2 RBD (Wild-Type) Antibody (IgG) Detection Kits (Vazyme, catalogue number DD3201-01), SARS-CoV-2 RBD (Omicron BA.4/5) Antibody (IgG) Detection Kits (Vazyme, catalogue number DD3214-01, China) and Mouse Anti-2019-nCoV (S) IgA Elisa Kits (FineTest, catalogue number 1906).

The S1 antibody assays were performed using a Mouse Anti-SARS-CoV-2 (B.1.1.529) Antibody IgG Titer Serologic Assay Kit (Spike S1) (Acro, catalogue number RAS-T061), Mouse Anti-SARS-CoV-2 Antibody IgG Titer Serologic Assay Kit (Spike S1) (Acro, catalogue number RAS-T045), Mouse Anti-SARS-CoV-2 (B.1.351) Antibody IgG Titer Serologic Assay Kit (Spike S1) (Acro, catalogue number RAS-T084), Mouse Anti-SARS-CoV-2 (B.1.617.2) Antibody IgG Titer Serologic Assay Kit (Spike S1) (Acro, catalogue number RAS-T086). The antibody serum samples that yielded optical density values at least 2.1-fold higher than that of the negative control were considered positive. The endpoint titre was defined as the highest serum dilution that yielded a positive optical density value. The geometric mean titre was calculated as the geometric mean of the endpoint titres of the positive serum samples in each group.

### Western blotting

Proteins were separated by 12% SDS–PAGE and transferred to polyvinylidene difluoride membranes. The membranes were blocked with 5% bovine serum albumin–Tris-buffered saline with Tween-20 (Sigma-Aldrich) and incubated with an anti-SARS-CoV-2 S1 antibody (MHC0102, Yunnan Lepeng Technology), anti-ACE2 antibody (Abcam, catalogue number ab15348), and anti-DC-SIGN antibody (Santa Cruz Biotechnology, catalogue number sc-74589) for 2 h; washed three times and incubated with HRP-conjugated goat anti-mouse IgG (H + L) (Sigma) for 1 h. Finally, the polyvinylidene difluoride membranes were washed three times and covered with ECL ultrasensitive chemiluminescence reagent (NCM Biotech, catalogue number P10100) and placed in a Bio-Rad gel imager for exposure and colour development.

### Silver staining with SDS‒PAGE

The prepared SDS–PAGE gel was silver-stained with a kit (Fast Silver Stain Kit, catalogue number P0017S, Beyotime). Silver staining of the SDS‒PAGE gels was performed in ten steps: fixation, washing with 30% ethanol, washing with water, sensitization, washing with water, silver staining, washing with water, colour development, termination and washing with water.

### Flow cytometry analysis

Lymph nodes and spleens were collected on days 3 and 7 after primary and booster immunization with the vaccine, and isolated lymphocyte. The flow-labelled antibodies used to detect the surface markers of DC activation were PE/Cy5-CD45 (catalogue number MA5-38732, Thermo Fisher), PE/Cy7-CD11c (catalogue number A15849, Thermo Fisher), FITC-CD80 (catalogue number A14722, Thermo Fisher), APC-CD83 (catalogue number ab234119, Abcam), and PE-CD86 (catalogue number 12-0862-82, Thermo Fisher). The flow-labelled antibodies used to detect specific T-cell surface markers were BV421-CD44 (catalogue number 103019, BioLegend), APC-CD25 (catalogue number 102011, BioLegend), PE/Cy5-CD3 (catalogue number 100205, BioLegend), FITC-CD4 (catalogue number 100405, BioLegend), APC/Cy7-CD8 (catalogue number 100713, BioLegend) and PE-Tetramer (Helixgen COVID-19 MHC-I Tetramer). The flow-labelled antibodies used to detect activated B-cell surface markers were FITC-CD19 (catalogue number 115505, BioLegend), PerCp/Cy5.5-GL7 (catalogue number 144609, BioLegend) and PE-S1 (Expedeon, catalogue number 336-005). The cells were stained for 30 min at 4 °C and washed twice prior to flow cytometric analysis (LSR Fortessa, BD).

### Luminex assays

The Bio-Plex Mouse 23-Plex Panel assay (catalogue number M60009RDPD) was performed according to the manufacturer’s instructions. Briefly, a standard curve ranging from 1.6 to 10,000 pg ml^−1^ was generated by serial dilution of the reconstituted standard. The filter plates were blocked by pipetting 200 µl of assay buffer into each well. After 10 min, the assay buffer was discarded by vacuum aspiration, and 25 µl of assay diluent was added to the wells designated for the samples, RPMI 1640 with GlutaMAX (Gibco) was added to the wells for the standards. Then, standard or sample was added to the appropriate wells, and 25 µl of antibody-coated fluorescent beads was added. Biotinylated secondary and streptavidin–phycoerythrin-labelled antibodies were subsequently added to the plate through alternating incubation and washing steps. Then, 100 µl of sheath fluid was added to the wells, and read immediately with the Bio-Plex array reader at high and low RP1 targets using a five-parameter logistic regression curve.

### Statistical analysis and reproducibility

For western blot and electron microscopy, immunofluorescence assays were repeated at least twice; for ELISA, quantitative analysis was repeated at least three times. All the data are expressed as mean values with s.e.m. Significant differences between groups were analysed by GraphPad Prism. Statistical significance was set to *P* < 0.05.

### Reporting summary

Further information on research design is available in the Nature Portfolio Reporting Summary linked to this article.

## Online content

Any methods, additional references, Nature Portfolio reporting summaries, source data, extended data, supplementary information, acknowledgements, peer review information; details of author contributions and competing interests; and statements of data and code availability are available at 10.1038/s41565-024-01679-1.

### Supplementary information

Supplementary Information Supplementary Figs. 1–10.

Reporting Summary

## Data Availability

The main data that support the findings of this study are available within the paper, via figshare at 10.6084/m9.figshare.25531513 (ref. ^29^) and the Supplementary Information. The transcriptomic data were uploaded to GSA (accession number CRA010542). Other raw and relevant data from the study are available for research purposes from the corresponding authors upon reasonable request.

## Extended data

**Extended Data Table 1:** Physicochemical characteristics of the LNP and VLS systems

Characterization	LNP	VLS
Size (nm)	81.41±1.2	114.99±2.54
Zeta potentials (mV)	34.5±1.274	32.97±1.03
PDI	0.19±0.01	0.16±0.02

The characteristics of the LNP and VLS system.

**Extended Data Table 2:** Symptoms of C57-hACE2 mice after SARS-CoV-2 infection Omicron BA.5

Group	(−)			VLS			mRNA			Peptide		
Symptom	3rd	5th	7th	3rd	5th	7th	3rd	5th	7th	3rd	5th	7th
Emaciation	−	+	++	−	−	−	−	−	−	−	−	+
Hollow back	−	+	++	−	−	−	−	−	+	−	−	+
Inverted hair	−	+	+	−	−	−	−	−	+	−	−	−
Poor appetite	+	+	++	−	−	−	−	−	−	−	−	+
Inactivity	−	+	++	−	−	−	−	−	−	−	−	−
**Omicron XBB.1**												
Group	(−)		VLS		mRNA		Peptide					
Symptom	2nd	5th	2nd	5th	2nd	5th	2nd	5th				
Emaciation	−	−	−	−	−	−	−	−/+				
Hollow back	−	−	−	−	−	−/+	−	−				
Inverted hair	−	+	−	−	−	−	−	−				
Poor appetite	+	+	−	−	−	−/+	−	−				
Inactivity	−	+	−	−	−	−	−	−/+				
**Wild type**												
Group	(−)		VLS		mRNA		Peptide					
Symptom	3rd	5th	3rd	5th	3rd	5th	3rd	5th				
Emaciation	−	+	−	−	−	−/+	−	−/+				
Hollow back	−	+	−	−	−	−	−	−				
Inverted hair	+	+	−	−	−	−	−	−				
Poor appetite	+	+	−	−	−	+	−	−				
Inactivity	+	++	−	−	−	+	−	+				

The symptoms of C57-hACE2 mice after SARS-CoV-2 infection

Omicron BA.5. ‘−’ represent no symptoms, ‘+’ represent milder symptoms, ‘++’ represent more obvious symptoms.

**Extended Data Table 3:** Histopathology of C57-hACE2 mice after SARS-CoV-2 infection

Omicron BA.5												
Group	(−)			VLS			mRNA			Peptide		
Tissue	3rd	5th	7th	3rd	5th	7th	3rd	5th	7th	3rd	5th	7th
Lung	++	++	++	+/−	−	−	+/−	+/−	−	+/−	+/−	+/−
Trachea	+	+	+	+/−	−	−	+/−	+/−	−	−	−	−
Heart	+/−	+/−	+/−	−	−	−	−	−	−	−	−	−
Liver	−	−	−	−	−	−	−	−	−	−	−	−
Stomach	−	−	−	−	−	−	−	−	−	−	−	−
Spleen	+/−	+/−	+/−	+/−	−	−	+/−	+/−		+/−	+/−	−
Spinal cord	+/−	+/−	+/−	−	−	−	−	−	−	−	−	−
Brain	−	−	−	−	−	−	−	−	−	−	−	−
Omicron XBB.1												
Group	(−)		VLS		mRNA		Peptide					
Tissue	2nd	5th	2nd	5th	2nd	5th	2nd	5th				
Lung	−	−	−	−	−	−	−	−/+				
Trachea	−	−	−	−	−	−/+	−	−				
Heart	−	+	−	−	−	−	−	−				
Liver	+	+	−	−	−	−/+	−	−				
Stomach	−	+	−	−	−	−	−	−/+				
Spleen	−	−	−	−	−	−	−	−				
Spinal cord	+	++	−/+	−/+	+	+	+	+				
Brain	+	++	−/+	−/+	+	+	+	+				
**Wild type**												
Group	(−)		VLS		mRNA		Peptide					
Tissue	3rd	5th	3rd	5th	3rd	5th	3rd	5th				
Lung	−	+	−	−	−	−/+	−	−/+				
Trachea	−	+	−	−	−	−	−	−				
Heart	+	+	−	−	−	−	−	−				
Liver	+	+	−	−	−	+	−	−				
Stomach	+	++	−	−	−	+	−	+				
Spleen	−	+	−	+/−	−	+	−	+				
Spinal cord	−/+	++	−/+	+	−/+	−/+	−/+	++				
Brain	+	++	−/+	−/+	+	++	+	−/+				

The histopathology of C57-hACE2 mice after SARS-CoV-2 infection. ‘−’ represents no histopathological changes were detected, ‘+’ represent the detection of lymphocyte infiltration; ‘++’ represents detection of lymphocyte infiltration and mild tissue damage.

**Extended Data Table 4:** Primers used for q-RT-PCR (Human)

Primer	Sequence(5′-3′)
RANKL-F	GGAGGAAGCACCAAGTATT
RANKL-R	CCTCTCCAGACCGTAACT
OX40L-F	GGTCAGGTCTGTCAACTCCTT
OX40L-R	CATCCAGGGAGGTATTGTCAGT
INFα-F	ACCCCTGCTATAACTATGACC
INFα-R	CTAACCACAGTGTAAAGGTGC
INFβ-F	AACTCCACCAGCAGACAG
INFβ-R	GAGAGCAGTTGAGGACATC
INFγ-F	ATGAACGCTACACACTGCATC
INFγ-R	CCATCCTTTTGCCAGTTCCTC
IKKα-F	ATACAGCGAGCAGATGAC
IKKα-R	CAACCTCAGCATAGTGGAT
IKKβ-F	CATTGTTGTTAGCGAAGACT
IKKβ-R	GCCAGGACACTGTTAAGAT
CD160-F	GCTGAGGGGTTTGTAGTGTTT
CD160-R	GTGTGACTTGGCTTATGGTGA
TLIA-F	AAGCCAGACTCCATCACT
TLIA -R	TACCTACTTCGCATACAGAC
NIK-F	CCGAGAAGAAGTCCACTG
NIK-R	GTCTGCTTGTCCTCCATC
4-1BBL-F	ACTCCTGGACTTAGACGAT
4-1BBL-R	GCTGGCACATTACAGATG
IL-13-F	CCTCATGGCGCTTTTGTTGAC
IL-13-R	TCTGGTTCTGGGTGATGTTGA
LIGHT-F	TCTTGCTGTTGTTCATTGC
LIGHT-R	CCTTCTTGGATGCTTCATTC
CXCL10-F	GGTGAGAAGAGATGTCTGAATCC
CXCL10-R	GTCCATCCTTGGAAGCACTGCA
CD209-F	GCAGTCTTCCAGAAGTAACCGC
CD209-R	GCTCTCCTCTGTTCCAATACTGC
CD160–F	GACCTACCAGTGTTGTGCCAGA
CD160-R	ATCCCGTCACTGTGTAGTTCCC
CCL18-F	GTTGACTATTCTGAAACCAGCCC
CCL18-R	GTCGCTGATGTATTTCTGGACCC
IL-33-F	GTGACGGTGTTGATGGTAAGAT
IL-33-R	AGCTCCACAGAGTGTTCCTTG
CCL16-F	GTGTTGCCAAGGAGACTAGTGG
CCL16-R	TCTTGGACCCAGTCGTCATTGG
IRF5-F	TATGCCATCCGCCTGTGTCAGT
IRF5-R	GCCCTTTTGGAACAGGATGAGC
STAT6-F	CCTTGGAGAACAGCATTCCTGG
STAT6-R	GCACTTCTCCTCTGTGACAGAC
CD163-F	CCAGAAGGAACTTGTAGCCACAG
CD163-R	CAGGCACCAAGCGTTTTGAGCT
TNFα-F	GTGAGGAGGACGAACATC
TNFα-R	TGAGCCAGAAGAGGTTGA

The primers used for q-RT-PCR (Human).

**Extended Data Table 5:** Primers used for q-RT-PCR (Mouse)

Primer	Sequence(5′-3′)
GAPDH-F	AGGTCGGTGTGAACGGATTTG
GAPDH-R	TGTAGACCATGTAGTTGAGGTCA
INFα-F	CTTCCTCAGACTCATAACCT
INFα-R	AGTCCTTCCTGTCCTTCA
INFβ-F	GATGAACTCCACCAGCAGACAGTG
INFβ-R	CACCATCCAGGCGTAGCTGTTG
INFγ-F	ATCAGGCCATCAGCAACAACA
INFγ-R	CGTCTCACCTCAAACTTGGCA
TNFα-F	GCCAACGGCATGGATCTCAA
TNFα-R	TCTTGACGGCAGAGAGGAGG
CD160-F	CCTGAGACCAACTTAGAACA
CD160-R	ACACCAACTGAGATGACTT
BTLA-F	GCCAGGACAGGAGAGTTA
BTLA-R	CTTACACCAAGTCACATTAGG
GMCSF-F	GGCCTTGGAAGCATGTAGAGG
GMCSF-R	GGAGAACTCGTTAGAGACGACTT
IL-1β-F	TCGCAGCAGCACATCAACAAGAG
IL-1β-R	TGCTCATGTCCTCATCCTGGAAGG
IL-4-F	GTGAGCTCGTCTGTAGGGCT
IL-4-R	CCGCTTACCGATGAATCCAGG
IL-6-F	TAGTCCTTCCTACCCCAATTTCC
IL-6-R	TTGGTCCTTAGCCACTCCTTC
IL-8-F	GGTGATATTCGAGACCATTTACTG
IL-8-R	GCCAACAGTAGCCTTCACCCAT
IL-9-F	ATGTTGGTGACATACATCCTTGC
IL-9-R	GACGGTGGATCATCCTTCAG
IL-10-F	GCTCTTACTGACTGGCATGAG
IL-10-R	CGCAGCTCTAGGAGCATGTG
IL-12-F	CCTCCTGTGGGAGAAGCAGA
IL-12-R	CTTGAGCCTTTCAGGCGGAG
IL-15-F	GTAGGTCTCCCTAAAACAGAGGC
IL-15-R	TCCAGGAGAAAGCAGTTCATTGC
IL-17A-F	AGATTACTACAACCGATCCACCT
IL-17A-R	GGGGACAGAGTTCATGTGGTA
IL-22-F	GCTTGACAAGTCCAACTTCCA
IL-22-R	GCTCACTCATACTGACTCCGT
IL-23a-F	AATAATGTGCCCCGTATCCAGT
IL-23a-R	GCTCCCCTTTGAAGATGTCAG
CXCL9-F	CCTAGTGATAAGGAATGCACGATG
CXCL9-R	CTAGGCAGGTTTGATCTCCGTTC
CXCL10-F	ATCATCCCTGCGAGCCTATCCT
CXCL10-R	GACCTTTTTTGGCTAAACGCTTTC
CXCL12-F	CACTGCCTATGTCCTCTTC
CXCL12-R	ACTGTTCTCCTGCTCCTT
CCL21-F	GTGATGGAGGGGGTCAGGA
CCL21-R	GGGATGGGACAGCCTAAACT
CCR7-F	AGAGGCTCAAGACCATGACGGA
CCR7-R	TCCAGGACTTGGCTTCGCTGTA
CD80-F	CTGCAAAGGACTTCAGAAACCT
CD80-R	AGGCTTCACCTAGAGAACCGT
CD83-F	ACCGTGGTTCTGAAGGTGACAG
CD83-R	CCAGAGAGAAGAGCAACACAGC
CD86-F	GGTGGCCTTTTTGACACTCTC
CD86-R	TGAGGTAGAGGTAGGAGGATCTT
MHCI-F	CCTACCAGAGAATGATTGGCTG
MHCI-R	GCAACTCATGCAGGTTGGC
MHCII-F	AGTGCGACGAGAACGGTAAC
MHCII-R	CGTTGGGGAACACACACCA
MIF-F	GAACCGCAACTACAGTAAGCTGC
MIF-R	ACGTTGGCAGCGTTCATGTCGT
GATA3-F	CCAGTAACCCTCTAAAGCC
GATA3-R	TATCCAACCCTGACCTCC

The primers used for q-RT-PCR (Mouse).
