# Supplementary material for: Virus-like structures for combination antigen protein mRNA vaccination
Source: Nat Nanotechnol. 2024 May 27;19(8):1224–33. doi: 10.1038/s41565-024-01679-1 (PMC11329372; doi:10.1038/s41565-024-01679-1)
Supplement: Supplementary file 1 — Supplementary Figs. 1–10. [file 41565_2024_1679_MOESM1_ESM.pdf]

nature nanotechnology

Supplementary information

https://doi.org/10.1038/s41565-024-01679-1

Virus-like structures for combination antigen protein mRNA vaccination

In the format provided by the authors and unedited

Supplementary Information

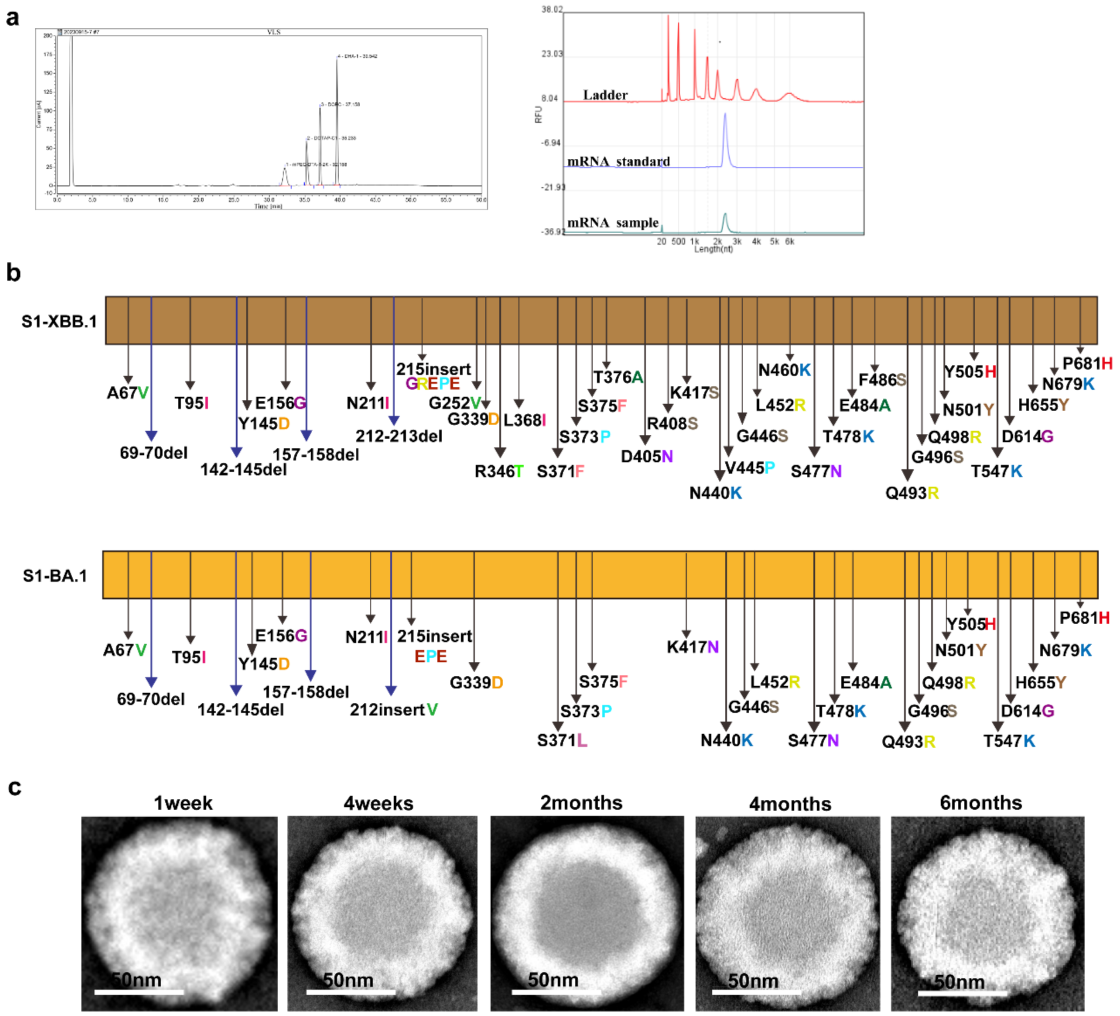

Supplementary Fig.1 Amino acid mutation sites of the Omicron XBB.1 strain and BA.1 strain, and electron micrographs of VLSs at different time points.

(a) Content of different lipids and molecular weight of mRNA in VLS vaccines. (b) The different colors indicate different amino acid mutations; Scale bars = 50 nm. (c) The electron micrographs of VLS at different time points. Experiments in figures a and c were replicated twice.

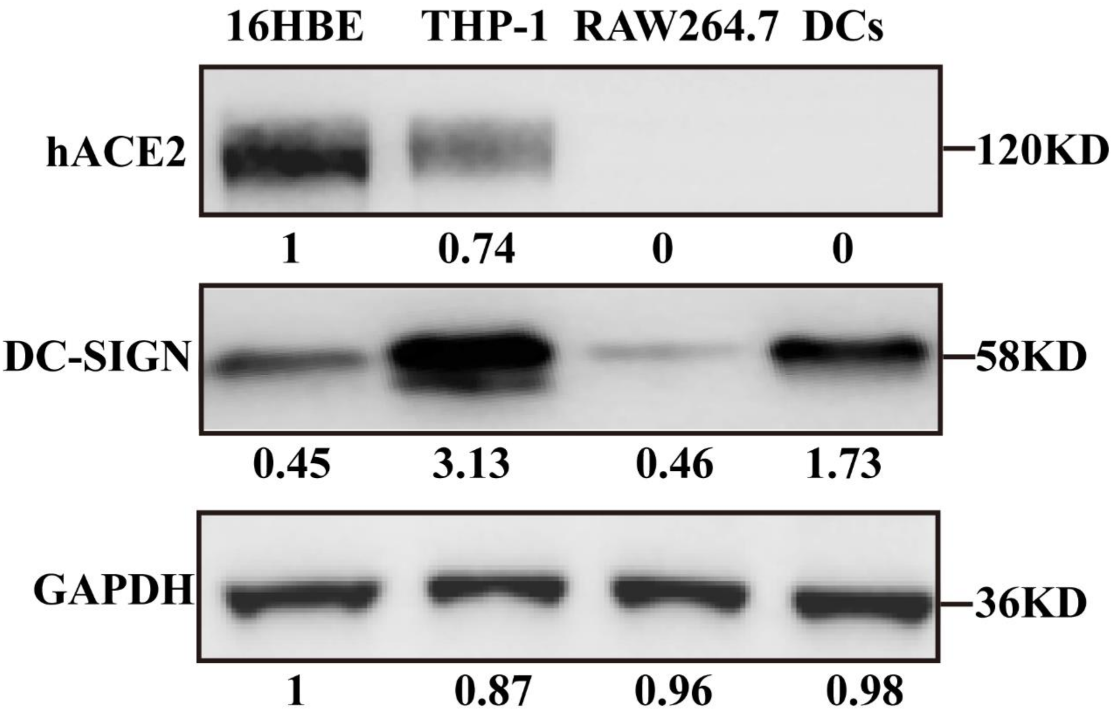

Supplementary Fig.2 Expression of hACE2 and DC-SIGN receptors on 16HBE, THP-1, RAW264.7 and DC cell lines. GAPDH was included as a protein loading control. The intensities of the signals (below the band) on Western blots were quantified by densitometric analysis with ImageJ (V1.8.0.112). Experiments of western blots were replicated twice.

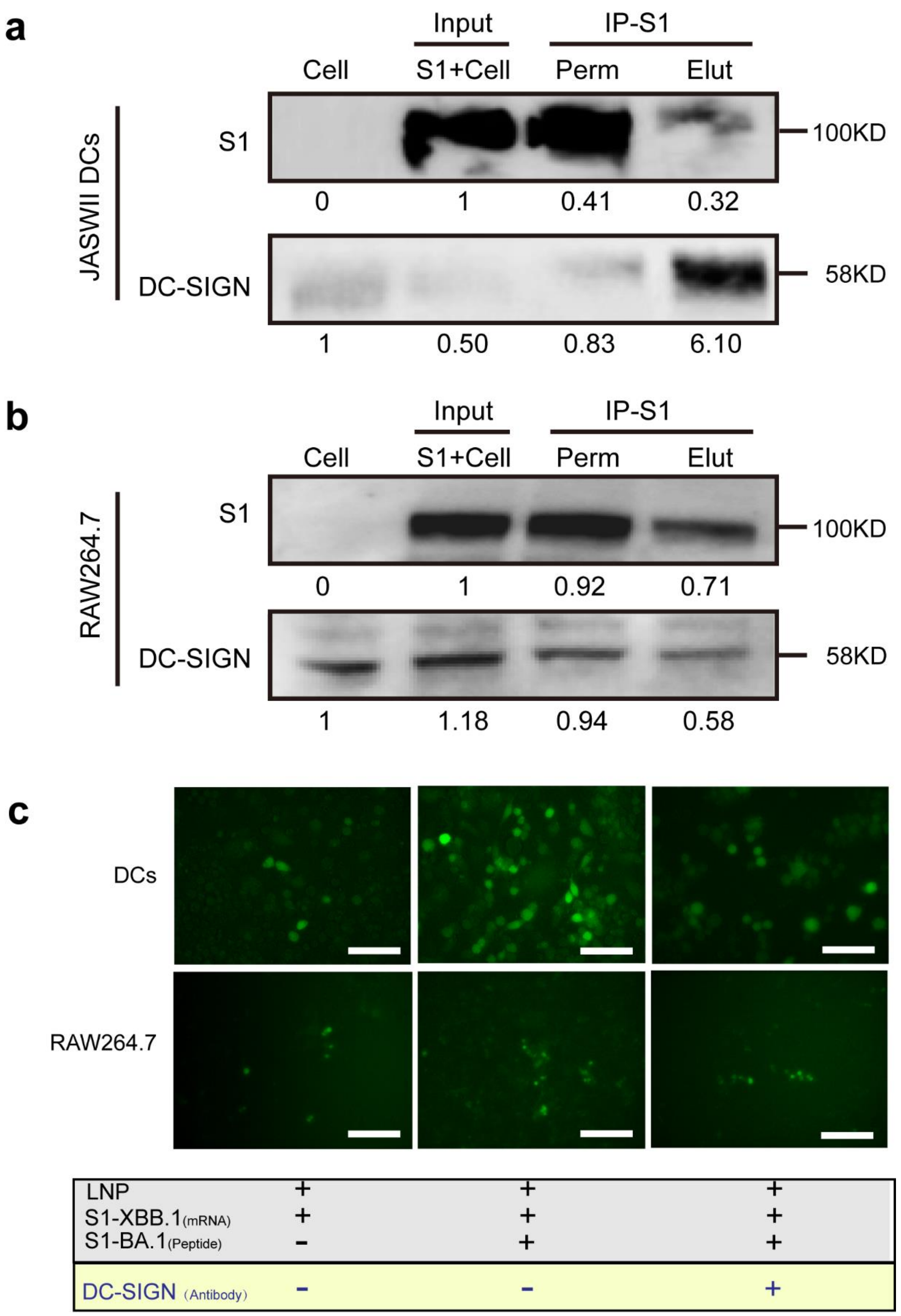

Supplementary Fig.3 The DC-SIGN receptor of DCs and RAW264.7 cells interacts with the S1 peptide of the SARS-CoV-2 Omicron BA.1 strain and VLSs enhance the transfection of cells to achieve mRNA delivery through the targeting of ACE2/DC-SIGN molecules via the S1 protein. The Omicron BA.1 strain S1 peptide

binds to DC-SIGN receptors of (a) DCs and (b) RAW264.7 cells. DC-SIGN receptors could be detected in the eluate from the IP experiments by Western blotting. The experimental loading volumes were equal and identified by Western blotting. (c) LNPs and VLSs encapsulating EGFP mRNA were transfected into DCs and RAW264.7 cells to obtain significant EGFP expression, the cells were treated with anti-DC-SIGN antibody, scale bars = 250 μm. Experiments of figure a and b were replicated twice, figure c was replicated three times.

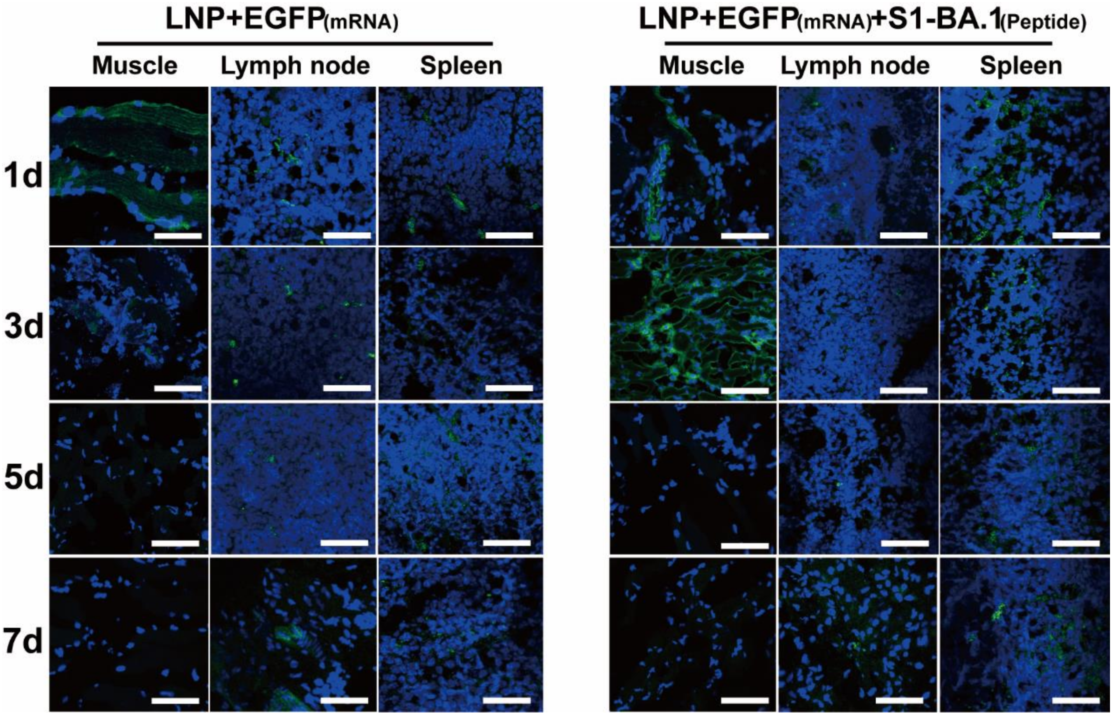

Supplementary Fig.4 Locations of EGFP expression of the mice.

Locations of EGFP expression in the muscles, lymph nodes and spleens of mice injected i.m. with either LNPs encapsulating EGFP mRNA or VLSs (LNPs encapsulating EGFP mRNA and S1 peptide of the BA.1 strain) on days 1, 3, 5 and 7. Scale bars = 500 μm. Experiment was replicated three times.

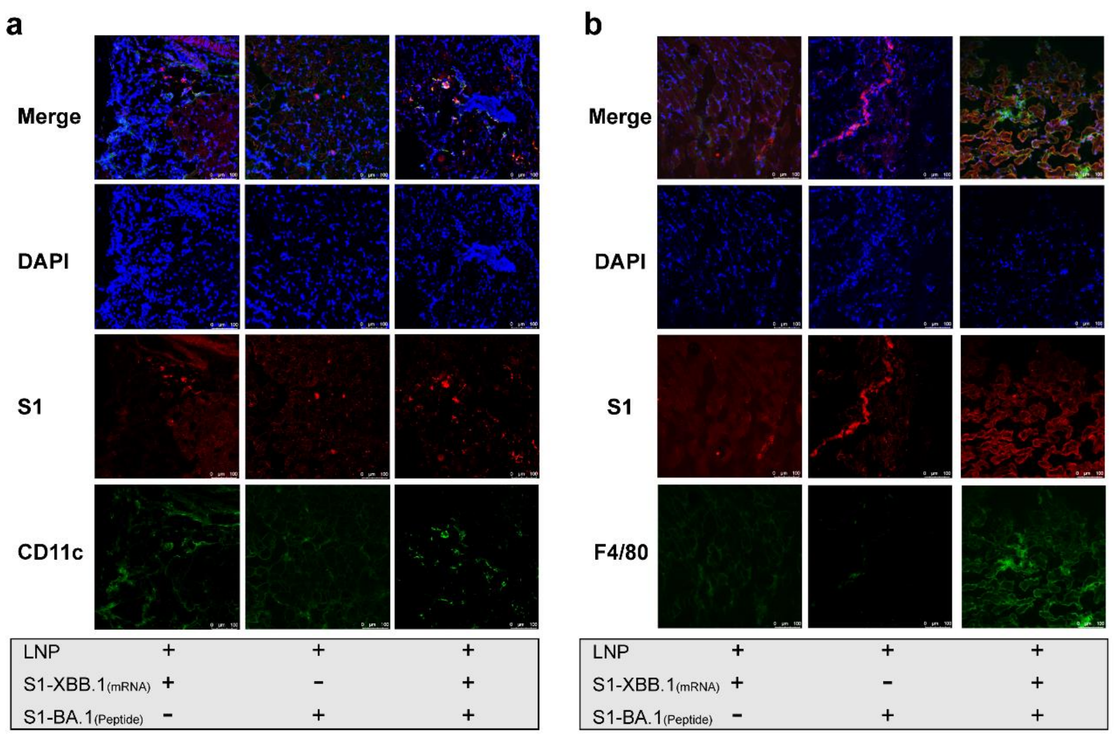

Supplementary Fig. 5 Colocalization of DCs or macrophages and S1 antigen in local tissues of mice immunized with VLSs, mRNA and peptide. Representative confocal fluorescence images of S1 expression (red) and specific cells (green) of mice injected i.m. with VLSs (LNPs encapsulating S1 mRNA of the XBB.1 strain and S1 peptide of the BA.1 strain), LNPs encapsulating S1 peptide (BA.1 strain), or LNPs encapsulating S1 mRNA (XBB.1 strain). (a) DCs marked by anti-CD11c antibodies or (b) macrophages marked by anti-F4/80 antibodies. Samples were obtained at 24, 48 and 72 h postimmunization (n = 3).

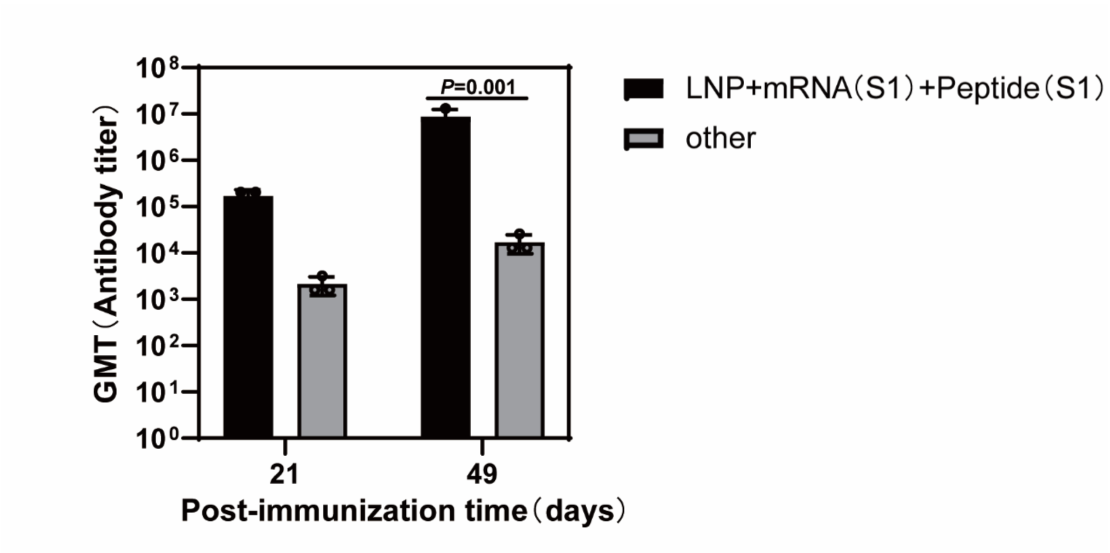

Supplementary Fig. 6 Titer of specific anti-Omicron strain in comparison with VLSs and the currently used vaccine. Antibodies whose production was induced by VLSs (LNPs encapsulating S1 mRNA of the XBB.1 strain and S1 peptide of the BA.1 strain) and other mRNA vaccines were used. This mRNA vaccine (China) has completed a phase III clinical trial. The data are shown as the means ± s.d. from three independent experiments (n = 3). Significant differences were determined using One-way ANOVA with Tukey’s post-hoc test.

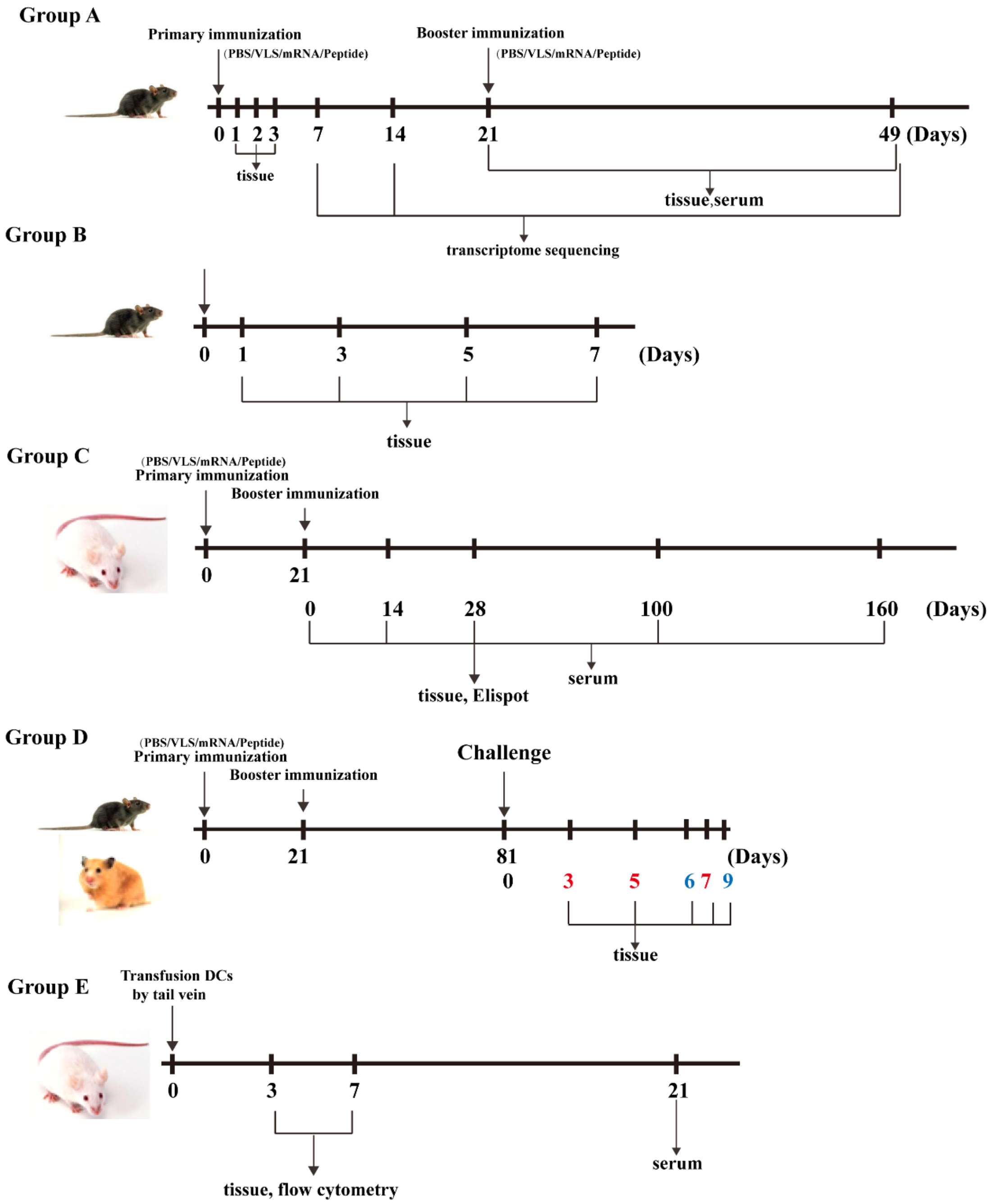

Supplementary Fig.7 Design of the animal experiment. The animal experimental

design consisted of five groups, A, B, C, D and E, as described in detail in the Methods section. Three mice or golden hamsters were euthanized at each time point to obtain tissues.

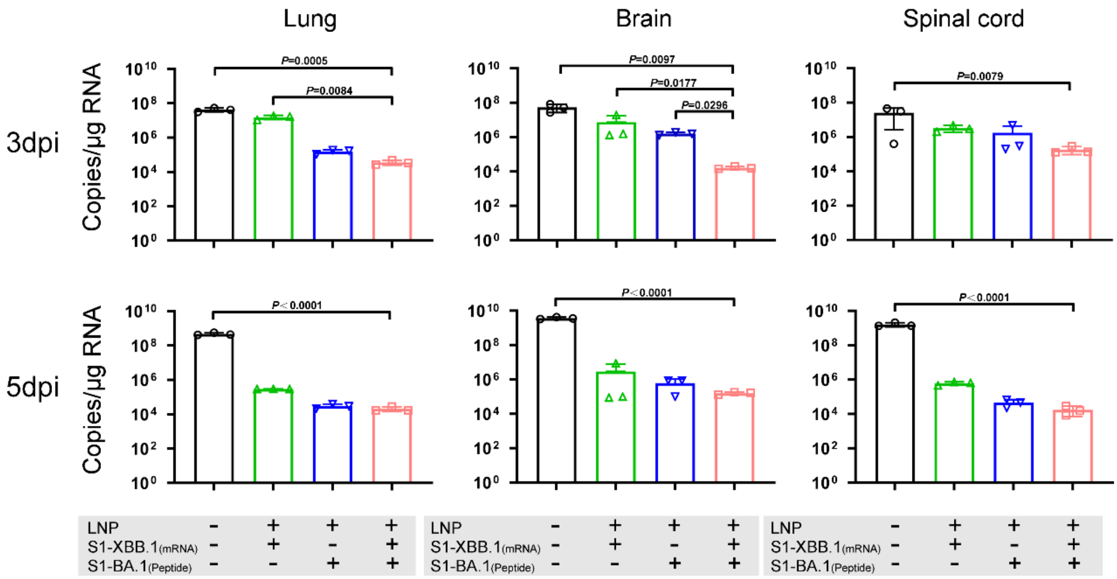

Supplementary Fig. 8 Effects of VLSs against WT strain challenge in C57-hACE2 mice. Viral loads in the lungs, brains and spinal cords of hACE2$^{+/+}$ mice injected i.m. with VLSs (LNPs encapsulating S1 mRNA of the XBB.1 strain and S1 peptide of the BA.1 strain), LNPs encapsulating S1 peptide (BA.1 strain), or LNPs encapsulating S1 mRNA (XBB.1 strain) were evaluated following infection with the WT strain, as determined by RT–qPCR. The data are shown as the means ± s.d. from three independent experiments (n = 3). Significant differences were determined using One-way ANOVA with Tukey's post-hoc test.

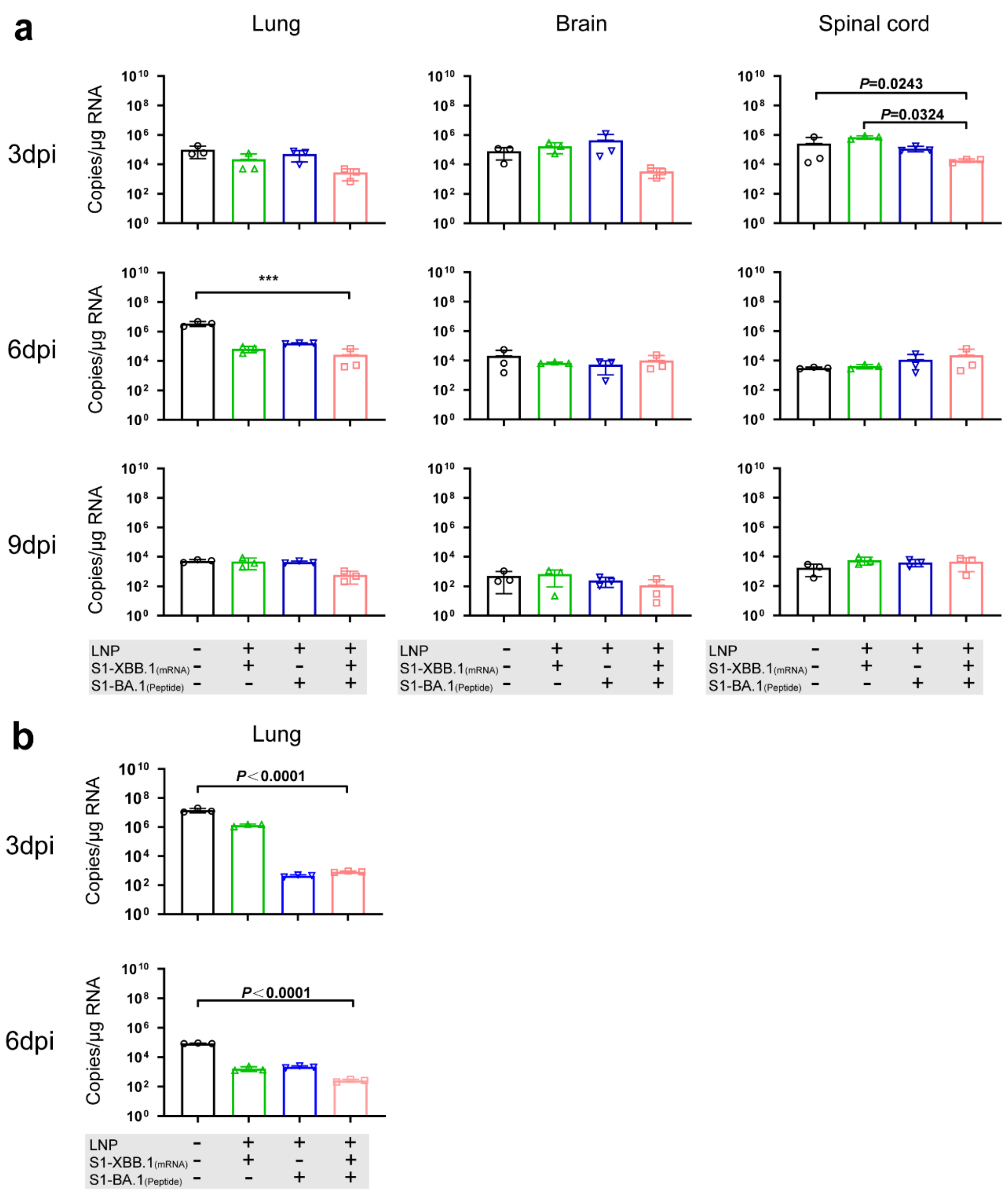

Supplementary Fig. 9 Effects of VLSs against challenge with the Omicron BA.5 strain or WT strain in hamsters. The viral loads in the lungs, brains and spinal cords of hamsters injected i.m. with VLSs (LNPs encapsulating S1 mRNA of the XBB.1 strain and S1 peptide of the BA.1 strain), LNPs encapsulating S1 peptide (BA.1 strain), or LNPs encapsulating S1 mRNA (XBB.1 strain) were evaluated following infection with the Omicron BA.5 (a) or WT stain (b), as determined by RT–qPCR. The data are shown as the means ± s.d. from three independent experiments (n = 3). Significant differences were determined using One-way ANOVA with Tukey's post-hoc test.

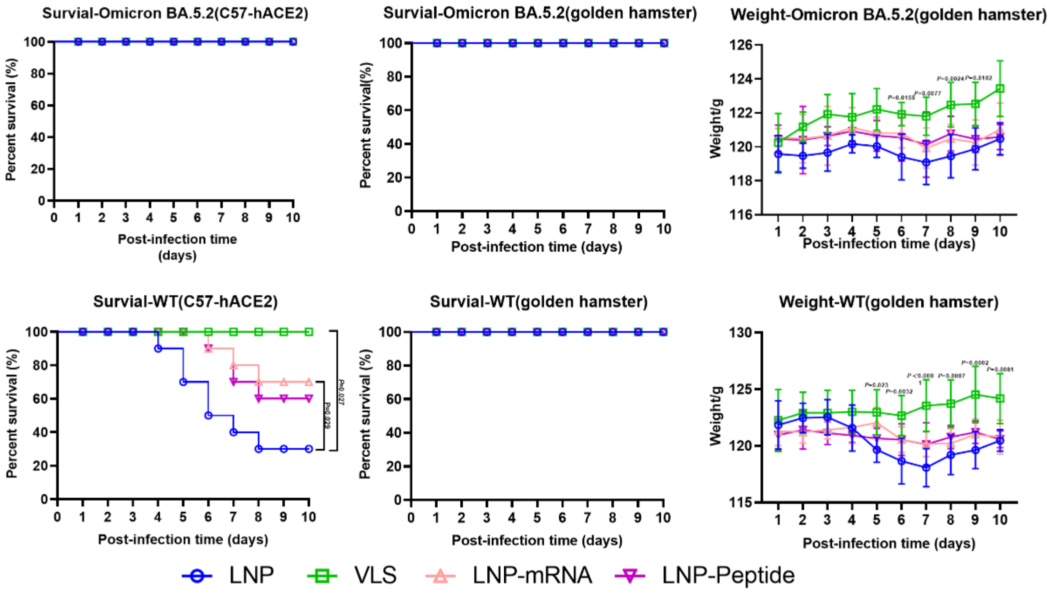

Supplementary Fig.10 Clinical manifestations of mice and hamsters after challenge with the Omicron BA.5 or WT strain. Four groups of mice and hamsters were injected i.m. with VLSs (LNPs encapsulating S1 mRNA of the XBB.1 strain and S1 peptide of the BA.1 strain), LNPs encapsulating S1 peptide (BA.1 strain), or LNPs encapsulating S1 mRNA (XBB.1 strain). (a) Survival of mice and hamsters after challenge with the Omicron BA.5 or WT strain. (b) Weight of hamsters after challenge with the Omicron BA.5 or WT strain. The data are shown as the means ± s.d. from three independent experiments (n = 4). Significant differences of mice survivals were determined using Gehan-Wilcoxon test, the wight of mice were determined using two-way ANOVA with Šidák's multiple-comparison test.
