# Supplementary material for: Virus-like structures for combination antigen protein mRNA vaccination
Source: Nat Nanotechnol. 2024 May 27;19(8):1224–33. doi: 10.1038/s41565-024-01679-1 (PMC11329372; doi:10.1038/s41565-024-01679-1)
Supplement: Supplementary file 2 — Reporting Summary [file 41565_2024_1679_MOESM2_ESM.pdf]

nature portfolio

Corresponding author(s): Qihan Li, Kaili Ma
Last updated by author(s): Apr 3, 2024

Reporting Summary

Nature Portfolio wishes to improve the reproducibility of the work that we publish. This form provides structure for consistency and transparency in reporting. For further information on Nature Portfolio policies, see our Editorial Policies and the Editorial Policy Checklist.

Statistics

For all statistical analyses, confirm that the following items are present in the figure legend, table legend, main text, or Methods section.

n/a	Confirmed	
☐	☒	The exact sample size (*n*) for each experimental group/condition, given as a discrete number and unit of measurement
☐	☒	A statement on whether measurements were taken from distinct samples or whether the same sample was measured repeatedly
☐	☒	The statistical test(s) used AND whether they are one- or two-sided<br>*Only common tests should be described solely by name; describe more complex techniques in the Methods section.*
☐	☒	A description of all covariates tested
☐	☒	A description of any assumptions or corrections, such as tests of normality and adjustment for multiple comparisons
☐	☒	A full description of the statistical parameters including central tendency (e.g. means) or other basic estimates (e.g. regression coefficient) AND variation (e.g. standard deviation) or associated estimates of uncertainty (e.g. confidence intervals)
☐	☒	For null hypothesis testing, the test statistic (e.g. *F*, *t*, *r*) with confidence intervals, effect sizes, degrees of freedom and *P* value noted<br>*Give P values as exact values whenever suitable.*
☐	☒	For Bayesian analysis, information on the choice of priors and Markov chain Monte Carlo settings
☐	☒	For hierarchical and complex designs, identification of the appropriate level for tests and full reporting of outcomes
☒	☐	Estimates of effect sizes (e.g. Cohen's *d*, Pearson's *r*), indicating how they were calculated

Our web collection on statistics for biologists contains articles on many of the points above.

Software and code

Policy information about availability of computer code

Data collection	No software uesd.
Data analysis	All statistical analyses were performed on GraphPad Prism v.9.0. Image J was used for image analysis. FlowJo(y.10) was used for flow cytometry analysis.

For manuscripts utilizing custom algorithms or software that are central to the research but not yet described in published literature, software must be made available to editors and reviewers. We strongly encourage code deposition in a community repository (e.g. GitHub). See the Nature Portfolio guidelines for submitting code & software for further information.

Data

Policy information about availability of data

All manuscripts must include a data availability statement. This statement should provide the following information, where applicable:

- Accession codes, unique identifiers, or web links for publicly available datasets
- A description of any restrictions on data availability
- For clinical datasets or third party data, please ensure that the statement adheres to our policy

The data that support the findings of this study are available within the paper. Source data are available for Figs.6. Additional materials from this study are available from the corresponding author on reasonable request. Source data are provided with this paper. https://ngdc.cncb.ac.cn/gsa/browse/cRA010542

Human research participants

Policy information about studies involving human research participants and Sex and Gender in Research.

Reporting on sex and gender	There are no human related research
Population characteristics	There are no human related research
Recruitment	There are no human related research
Ethics oversight	There are no human related research

Note that full information on the approval of the study protocol must also be provided in the manuscript.

Field-specific reporting

Please select the one below that is the best fit for your research. If you are not sure, read the appropriate sections before making your selection.

☒ Life sciences ☐ Behavioural & social sciences ☐ Ecological, evolutionary & environmental sciences

For a reference copy of the document with all sections, see nature.com/documents/nr-reporting-summary-flat.pdf

Life sciences study design

All studies must disclose on these points even when the disclosure is negative.

Sample size	The sample size was calculated based on the different time points and the number of each time point, Sample sizes of in vitro,ex vivo and in vivo experiments were determined according to previous experimental experience or previously published literature.
Data exclusions	No data were excluded from the analyses.
Replication	All experimental data are even includine replicates. Details of experimental replicates are given in the figure legends.
Randomization	All samples/organisms were randomly allocated into experimental groups.
Blinding	The investigators were blinded to group allocation during data collection or analysis

Reporting for specific materials, systems and methods

We require information from authors about some types of materials, experimental systems and methods used in many studies. Here, indicate whether each material, system or method listed is relevant to your study. If you are not sure if a list item applies to your research, read the appropriate section before selecting a response.

Materials & experimental systems

n/a	Involved in the study	
☐	☒	Antibodies
☐	☒	Eukaryotic cell lines
☒	☐	Palaeontology and archaeology
☐	☒	Animals and other organisms
☒	☐	Clinical data
☐	☒	Dual use research of concern

Methods

n/a	Involved in the study	
☒	☐	ChIP-seq
☐	☒	Flow cytometry
☒	☐	MRI-based neuroimaging

Antibodies

Antibodies used

anti-SARS-CoV-2 S1antibody(MHC0102, Yunnan Lepeng Technology Co., Ltd),
anti-ACE2 antibody(Abcam, Cat# ab15348, polyclona),
anti DC-SIGN antibody(Santa Cruz Biotechnology, Cat# sc-74589,clobe:-2),
HRP-conjugated goat anti-mouse IgG(H+L)(Sigma, Shanghai, China,Cat# AP132,polyclona),
anti-CD11c antibody (Abcam, UK, Cat# ab33483,clone:N418);
anti-F4/80 antibody (Zhengneng, China, Cat# 263101,clone:31B1);
Alexa Fluor 647-conjugated goat anti mouse IgG (Thermofisher, Cat # A32728,Polyclonal);

Alexa Fluor 488-conjugated goat anti-rabbit IgG (Thermofisher,A32731,Polyclonal);
PE/Cy5-CD45 (Cat# MA5-38732,clone:2D1, Thermo Fisher),;
PE/Cy7-CD11c (Cat# A15849, clone:N418,Thermo Fisher);
FITC-CD80 (Cat# A14722, clone:1G10,Thermo Fisher);
APC-CD83 (Cat# ab234119, clone:HB15e,Abcam);
PE-CD86 (Cat# 12-0862-82,clone:GL1, Thermo Fisher);
BV421-CD44 (Cat# 103019, clone:IM7,BioLegend),
APC-CD25 (Cat# 102011, clone:PC61,BioLegend),
PE/Cy5-CD3 (Cat# 100205, clone:17A2,BioLegend),
FITC-CD4 (Cat# 100405, clone:GK.1,BioLegend),
APC/Cy7-CD8 (Cat# 100713, clone:53-6.7,BioLegend);
FITC-CD19 (Cat# 115505, clone:6D5,BioLegend),
PerCp/Cy5.5-GL7 (Cat# 144609, clone:GL7,BioLegend);

Validation

anti-SARS-CoV-2 S1antibody(MHC0102, Yunnan Lepeng Technology Co., Ltd), this antibody is customized
anti-ACE2 antibody(Abcam, Cat# ab15348, polyclona), https://www.abcam.cn/products/primary-antibodies/ace2-antibody-ab15348.html;
anti DC-SIGN antibody(Santa Cruz Biotechnology, Cat# sc-74589,clobe:-2),https://www.scbt.com/zh/p/dc-sign-dc-signr-antibody-b-2;
HRP-conjugated goat anti-mouse IgG(H+L)(Sigma, Shanghai, China,Cat# AP132,polyclona),https://www.sigmaaldrich.cn/CN/zh/product/mm/ap132;
anti-CD11c antibody (Abcam, UK, Cat# ab33483,clone:N418),https://www.abcam.cn/products/primary-antibodies/cd11c-antibody-n418-ab33483.html;
anti-F4/80 antibody (Zhengneng, China, Cat# 263101,clone:31B1),http://www.zen-bio.cn/products.aspx?FId=t3:1:3&TypeId=1&IsActiveTarget=True&key=263101&t=0.4047531853461257;
Alexa Fluor 647-Goat anti mouse IgG (Thermofisher, Cat # A32728),https://www.thermofisher.cn/order/genome-database/browse/antibody/sub-type/antibody_secondary/keyword/alexa%20647;
Alexa Fluor 488-conjugated goat anti-rabbit IgG (Thermofisher,A32731,Polyclonal),https://www.thermofisher.cn/cn/zh/antibody/product/Goat-anti-Rabbit-IgG-H-L-Highly-Cross-Adsorbed-Secondary-Antibody-Polyclonal/A32731;
PE/Cy5-CD45 (Cat# MA5-38732,clone:2D1, Thermo Fisher), https://www.thermofisher.cn/cn/zh/antibody/product/CD45-Antibody-clone-2D1-Monoclonal/MA5-38732;
PE/Cy7-CD11c (Cat# A15849, clone:N418,Thermo Fisher),https://www.thermofisher.cn/cn/zh/antibody/product/CD11c-Antibody-clone-N418-Monoclonal/A15849;
FITC-CD80 (Cat# A14722, clone:1G10,Thermo Fisher), https://www.thermofisher.cn/cn/zh/antibody/product/CD80-Antibody-clone-1G10-Monoclonal/A14722;
APC-CD83 (Cat# ab234119, clone:HB15e,Abcam), https://www.abcam.cn/products/primary-antibodies/apc-cd83-antibody-hb15e-ab234119.html;
PE-CD86 (Cat# 12-0862-82, clone:GL1,Thermo Fisher),https://www.thermofisher.cn/cn/zh/antibody/product/CD86-B7-2-Antibody-clone-GL1-Monoclonal/12-0862-82;
BV421-CD44 (Cat# 103019, clone:IM7,BioLegend), https://www.biolegend.com/en-gb/products/pacific-blue-anti-mouse-human-cd44-antibody-3099;
APC-CD25 (Cat# 102011, clone:PC61,BioLegend), https://www.biolegend.com/en-gb/products/apc-anti-mouse-cd25-antibody-420
PE/Cy5-CD3 (Cat# 100205, clone:17A2,BioLegend), https://www.biolegend.com/en-gb/products/pe-anti-mouse-cd3-antibody-47;
FITC-CD4 (Cat# 100405, clone:GK.1,BioLegend), https://www.biolegend.com/en-gb/products/fitc-anti-mouse-cd4-antibody-248
APC/Cy7-CD8 (Cat# 100713, clone:53-6.7,BioLegend),https://www.biolegend.com/en-gb/products/apc-cyanine7-anti-mouse-cd8a-antibody-2269;
FITC-CD19 (Cat# 115505, clone:6D5,BioLegend), https://www.biolegend.com/en-gb/products/fitc-anti-mouse-cd19-antibody-1528
PerCp/Cy5.5-GL7 (Cat# 144609, clone:GL7,BioLegend), https://www.biolegend.com/en-gb/products/percp-cyanine5-5-anti-mouse-human-gl7-antigen-t-and-b-cell-activation-marker-antibody-9231;

Eukaryotic cell lines

Policy information about cell lines and Sex and Gender in Research

Cell line source(s)

HEK-293T(CRL-3216M)is an epithelial-like cell that was isolated from the kidney of a patient, this cell line was deposited by Stanford University and can be used in vaccine development; JASWll dendritic cells with a monocyte morphology cell that was isolated from the bone marrow of a mouse, this cell line was deposited by ZymoGenetics Incorporation,this product is an ATCC manufactured and accessioned progeny of ATCC CRL-11904cited in US Pat.No.5,648,219;RAW 264.7(TIB-71m)is a macrophage cell line that was established from a tumor in a male mouse induced with the Abelson murine leukemia virus;THP-1(TIB-202 m) is a monocyte isolated from peripheral blood from an acute monocytic leukemia patient;16HBE (CRL-2078 mM) is a epithelial cell from human. All the cells purchased from ATCC.

Authentication

The cell lines were not authenticated.

Mycoplasma contamination

All cell lines tested negative for mycoplasma contamination

Commonly misidentified lines
(See ICLAC register)

No commonly misidentified cell lines were used.

Animals and other research organisms

Policy information about studies involving animals; ARRIVE guidelines recommended for reporting animal research, and Sex and Gender in Research

Laboratory animals	Balb/c mice, 4-5 weeks; Human AcE2 gene knock-in mice were constructed on the C57BL/6 background 4-5 weeks; Golden hamsters, 7-8 weeks. Animals were housed in groups of 4-6 mice perindividually ventilated cage in a 12 h light dark cycle (06:30-18:30 light; 18:30-06:30 dark), with constant room temperature (22 ±1?) and relative humidity(40-60 %). Animals had access to food and water ad libitum.
Wild animals	The study did not involve wild animals.
Reporting on sex	All animal experiments were performed using female mice, which did not affect the results given their easy grasp.
Field-collected samples	All collected animal tissues were stored in triplicate and stored in a -80 ° C refrigerator, and all isolated animal sera were stored in a -20? refrigerator. The tissues involved in this study were all preserved, and no tissues that were not preserved were involved?
Ethics oversight	Animal Experiment Ethics Committee of Shandong WeigaoLitong Biological Products Co., Ltd. (approval number: LACUC-RD3-2022-006)

Note that full information on the approval of the study protocol must also be provided in the manuscript.

Dual use research of concern

Policy information about dual use research of concern

Hazards

Could the accidental, deliberate or reckless misuse of agents or technologies generated in the work, or the application of information presented in the manuscript, pose a threat to:

No	Yes	
☒	☐	Public health
☒	☐	National security
☒	☐	Crops and/or livestock
☒	☐	Ecosystems
☒	☐	Any other significant area

Experiments of concern

Does the work involve any of these experiments of concern:

No	Yes	
☒	☐	Demonstrate how to render a vaccine ineffective
☒	☐	Confer resistance to therapeutically useful antibiotics or antiviral agents
☒	☐	Enhance the virulence of a pathogen or render a nonpathogen virulent
☒	☐	Increase transmissibility of a pathogen
☒	☐	Alter the host range of a pathogen
☒	☐	Enable evasion of diagnostic/detection modalities
☒	☐	Enable the weaponization of a biological agent or toxin
☒	☐	Any other potentially harmful combination of experiments and agents

Flow Cytometry

Plots

Confirm that:

☒ The axis labels state the marker and fluorochrome used (e.g. CD4-FITC).

☒ The axis scales are clearly visible. Include numbers along axes only for bottom left plot of group (a 'group' is an analysis of identical markers).

☒ All plots are contour plots with outliers or pseudocolor plots.

☒ A numerical value for number of cells or percentage (with statistics) is provided.

Methodology

Sample preparation	PBMC were obtained from mouse spleen or lymph node tissue through lymphocyte separation medium and then washed with PBS before antibody labeling.
Instrument	flow cytometric analysis (LSR Fortessa, BD)
Software	FlowJo
Cell population abundance	The purity of PBMC was more than 90%.
Gating strategy	The control group and the experimental group were detected respectively, and the positive and negative gates were divided according to the labeled antibody and non-labeled antibody in the experimental group.

☒ Tick this box to confirm that a figure exemplifying the gating strategy is provided in the Supplementary Information.
